# RNA sequencing reveals potential interacting networks between the altered transcriptome and ncRNome in the skeletal muscle of diabetic mice

**DOI:** 10.1042/BSR20210495

**Published:** 2021-07-12

**Authors:** Devesh Kesharwani, Amit Kumar, Mukta Poojary, Vinod Scaria, Malabika Datta

**Affiliations:** 1 CSIR-Institute of Genomics and Integrative Biology, Functional and Genomics Unit, Mall Road, Delhi, India; 2 Academy of Scientific and Innovative Research, CSIR-HRDC, Kamala Nehru Nagar, Ghaziabad 201002, Uttar Pradesh, India; 3 GN Ramachandran Knowledge Centre for Genome Informatics, CSIR-Institute of Genomics and Integrative Biology, Mathura Road, Delhi 110025, India

**Keywords:** diabetes, ncRNA, RNA sequencing, skeletal muscle

## Abstract

For a global epidemic like Type 2 diabetes mellitus (T2DM), while impaired gene regulation is identified as a primary cause of aberrant cellular physiology; in the past few years, non-coding RNAs (ncRNAs) have emerged as important regulators of cellular metabolism. However, there are no reports of comprehensive in-depth cross-talk between these regulatory elements and the potential consequences in the skeletal muscle during diabetes. Here, using RNA sequencing, we identified 465 mRNAs and 12 long non-coding RNAs (lncRNAs), to be differentially regulated in the skeletal muscle of diabetic mice and pathway enrichment analysis of these altered transcripts revealed pathways of insulin, FOXO and AMP-activated protein kinase (AMPK) signaling to be majorly over-represented. Construction of networks showed that these pathways significantly interact with each other that might underlie aberrant skeletal muscle metabolism during diabetes. Gene–gene interaction network depicted strong interactions among several differentially expressed genes (DEGs) namely, *Prkab2, Irs1, Pfkfb3, Socs2* etc. Seven altered lncRNAs depicted multiple interactions with the altered transcripts, suggesting possible regulatory roles of these lncRNAs. Inverse patterns of expression were observed between several of the deregulated microRNAs (miRNAs) and the differentially expressed transcripts in the tissues. Towards validation, overexpression of miR-381-3p and miR-539-5p in skeletal muscle C2C12 cells significantly decreased the transcript levels of their targets, *Nfkbia, Pik3r1* and *Pi3kr1, Cdkn2d*, respectively. Collectively, the findings provide a comprehensive understanding of the interactions and cross-talk between the ncRNome and transcriptome in the skeletal muscle during diabetes and put forth potential therapeutic options for improving insulin sensitivity.

## Introduction

Type 2 diabetes mellitus (T2DM) is a leading global concern affecting almost 463 million people in the age group of 20–79 years worldwide [1]. Such alarming numbers are majorly attributed to physical inactivity accompanied by stress and obesity [2–6]. Aberrant glucose metabolism and homeostasis remain the major underlying events and the skeletal muscle plays a very significant role herein [7]. It is considered as the prime site for almost 80% of glucose disposal in the postprandial state and therefore is significant in the onset and progression of insulin resistance and diabetes [8]. Anomalous skeletal muscle metabolism is reported in various rodent models of diabetes as well as in human subjects [9–14]. Skeletal muscle is the predominant site for glycogen synthesis and under normal physiological conditions, majority (almost two-thirds) of the glucose taken up by the skeletal muscle cell gets converted into glycogen and the remaining enters the glycolytic cycle [7]. By far, the earliest detectable events of insulin resistance observed even years before the onset of T2DM is impaired glycogen synthesis in the skeletal muscle as evidenced by studies on normal glucose-tolerant subjects having type 2 diabetic parents [7,15,16]. Activation of certain protein kinases by intramyocellular fats and fatty acid metabolites has been strongly linked to impaired insulin signaling [17]. This increased intramyocellular fat content is, to some extent attributed to mitochondrial defects and impaired mitochondrial oxidative phosphorylation in insulin-resistant states [14]. Impaired muscle regeneration with reduced muscle cell plasticity, along with deleterious changes in satellite cell function are identified as underlying central mechanisms for the transition from a healthy muscle fiber to the diseased muscle physiology during diabetes [18].

Aberrant regulation and function of several genes and pathways are believed to be the underlying causes of such skeletal muscle dysfunction as observed in diabetic animal models and human subjects. These include but are not limited to IRS 1 [19], GLUT4 [20], glycogen synthetase [21], UCP-3 [22], PI3-Kinase [23], hexokinase II [24], MAPK [23], Rad genes [25], serine-threonine kinases [26], calpain-10 [27] etc. In addition, during the last few years, several species of non-coding RNAs (ncRNAs) have been identified to depict altered signatures in the skeletal muscle during diabetes [28–30], and these have provided new evidences on their important regulatory roles in this tissue.

Microarray and RNA-sequencing technologies have greatly aided in this by providing information of ncRNAs’ global cellular profile and status. These ncRNAs, by varied mechanisms and by affecting the levels and functions of various transcripts, play critical roles in aberrant skeletal muscle metabolism. In the recent past, long non-coding RNAs (lncRNAs) have emerged as critical players involved in the processes of myogenesis and muscle differentiation. The lncRNA, H19 has been shown to enhance muscle insulin sensitivity, at least in part, by activating AMP-activated protein kinase (AMPK) signaling [31]. Specifically expressed in the skeletal muscle, the lncRNA, MUNC regulates myogenesis by regulating the expression of the myogenic transcription factor, myoD [32]. Activated by myoD, lnc MyoD is critical in skeletal muscle regeneration and myogenesis [33]. Being epigenetically regulated by myoD, the lncRNA, Malat1 accelerates the process of muscle differentiation and improves muscle regeneration in Malat1 knockout and dystrophic mdx mice [34]. While few lncRNAs such as lnc-YY1 [35], lnc-Dum [36], lnc-Glt2/Meg3 [37], lnc-MD1 [38] and lnc-mg [39] positively regulate the process of myogenesis, others such as lncRNA H19 [40], lnc-31 [41], Yam (YY1-associated muscle lncRNAs) [42], Sirt1 AS [43] and Malat1 negatively influence the process of myogenesis [34].

Various studies show that another class of ncRNAs, the microRNAs (miRNAs) are critical players in the fine-tuning and regulation of transcriptional processes by maintaining optimum levels of various transcripts required by the cell. miRNA-mediated regulation of transcripts of critical genes of the insulin signaling cascade such as IRS1 by miR-128a [44] and miR-29 [45]; IRS-2 by miR-135a [29]; PTEN by miR-21 [46] and miR-494 [47]; IGF-1 by miR-126 [48] and miR-1 [49]; IGF-2R receptor and IGFBP5 by miR-143-3p [50]; PPARδ by miR-29a [51]; Hexokinase 2 (HK2), IRS-1, GLUT1, PIK3R3 and AKT2 by miR-29a and 29c [52]; GLUT4 by miR-17 [53] and miR-106b [54]; JAG1 by miR-449a [55] have been reported in diverse models of diabetes. These miRNA–mRNA interactions greatly affect pathways and consequently impair the respective metabolic processes within the cell.

All these data suggest that aberrant regulation of ncRNAs and mRNAs as seen during diabetes affect cellular metabolic pathways and muscle physiology. However, in spite of these independent studies, there is no evidence of comprehensive interactive networks between altered mRNAs and ncRNA signatures in the skeletal muscle during diabetes. In the present study, using normal (db/+) and diabetic (db/db) mice, we sought to explore the cross-talk between altered skeletal muscle mRNA transcripts and ncRNAs with an attempt to provide a comprehensive in-depth analysis of the aberrant physiology of the skeletal muscle during diabetes.

## Materials and methods

### Animal experiments

Ten-week-old normal (C57BLKs db/+) and diabetic (C57BLKs db/db) male mice (*n*=4) were obtained from CSIR-Central Drug Research Institute, Lucknow, India, kept in a 12:12-h light–dark cycle at the CSIR-Institute of Genomics and Integrative Biology, New Delhi (India) and given *ad libitum* food and water. Body weight and glucose (measured using ACCU-CHEK, Roche, Mannhein Germany) status of the animals taken for the study are given in Supplementary Figure S1. Mice were euthanized using sodium thiopentone and skeletal muscles were immediately isolated from both db/+ and db/db animals and stored in RNA later™ solution (Ambion Life Technologies, CA, U.S.A.) at −80°C until further use. The Institutional Animal Ethics Committee (IAEC) of CSIR-Institute of Genomics and Integrative Biology, New Delhi, India approved the animal experiments and procedures (IGIB/IAEC/OCT/2018/20), and they were performed according to the guidelines of the Committee for the Purpose of Control and Supervision of Experiments on Animals (CPCSEA), New Delhi, India. All experiments were performed at the Animal House Facility of CSIR-Institute of Genomics and Integrative Biology, New Delhi, India.

### RNA isolation and RNA sequence library preparation and data generation

Total RNA from skeletal muscles of normal and diabetic mice were isolated using the Ambion mirVana isolation kit (Ambion, Austin, TX, U.S.A.). RNA concentration was measured by Infinite 200 PRO plate reader (Tecan, Mannedorf, Switzerland) and the 260/280 ratio of isolated RNA was between 1.9 and 2.0. Total RNA (1 µg) was treated with Ribo-Zero Gold rRNA removal kit (Illumina Inc., CA, U.S.A.) and libraries were prepared using Truseq standard total RNA library preparation kit according to manufacturer’s instructions, which enriches libraries with polyA-tailed transcripts (Illumina Inc., CA, U.S.A.). All samples were sequenced by Illumina’s sequenced-by-synthesis technology, with 101-bp paired end sequencing in a HiSeq 4000 platform (Illumina Inc., CA, U.S.A.) by Bionivid Technology Pvt. Ltd., Bengaluru, India.

### RNA sequencing data analysis

The determination of read quality of the RNA-seq data was done using the FastQC (v0.11.7) program. The bad quality reads were trimmed using Trimmomatic program (v0.36) and the read quality was then re-examined with FastQC to confirm proper trimming of bad quality reads. Cleaned and quality checked reads were then mapped to the mouse reference genome GRCm38/M21 using HISAT2 [56] software (v 2.1.0) with default parameters that resulted in an average alignment percentage close to 96%. Two different tools, Cuffdiff and DESeq2 were used to minimize false positives while determining differential gene expression between normal and diabetic mice using the transcript model from GENCODE (M21 version). R was used to plot the volcano plot to visualize fold changes and *P*-values among all the identified transcripts in the skeletal muscle of db/db mice as compared with normal db/+ mice. Transcripts having a fold change of at least ≥ ±2 and *P*-value <0.01 were considered as differentially expressed genes (DEGs). Raw data of the sequenced reads from normal (db/+) and diabetic (db/db) mice livers have been deposited in the SRA with accession number SRP238785. All DEGs identified as significantly differentially expressed in both programs as stated above were considered for further downstream analysis. Visualization of fragments per kilobase of transcripts per million mapped reads (FPKM) values of DEGs in normal and diabetic mice was done using R package, pheatmap.

### Pathway enrichment and construction of gene–gene interaction network

Pathway enrichment analysis for DEGs was performed using DAVID Bioinformatics Resources [57] v6.8 (https://david.ncifcrf.gov/) applying Fisher’s exact test with *P*-value <0.01. Functional Analysis visualization was done using Cytoscape [58] and ClueGO [59]. The significantly enriched pathways with adjusted *P*-value <0.05 after Benjamini–Hochberg correction represented networks along with the genes involved in these pathways. For gene–gene interaction network analysis, read counts for all expressed genes (total transcripts detected) were estimated using HTSeq program [60]. These read counts were median normalized using R package EBSeq [61]. Correlation between each of these expressed genes was estimated using the R package, psych. The correlation was calculated using the Pearson method [62], its significance was evaluated, and corrected using Benjamini–Hochberg method. All expressed genes having correlations greater than ±0.98 and a significant *P*-value <0.05 are represented in a network.

### MiRNA target prediction

In a previous study, we had reported an altered miRNA profile in the skeletal muscle of diabetic db/db mice [29]. We extracted putative targets of these miRNAs from TargetScan [63] (http://www.targetscan.org/mmu_71/) and miRDB [64] (http://www.mirdb.org/) databases. Common targets to each miRNA from both tools were matched with DEGs as obtained in the present study to interrogate inverse patterns of expression of miRNAs and the DEGs.

### Cell culture

Inverse patterns of expression between the miRNAs and their respective predicted targets were validated in C2C12 skeletal muscle cells. C2C12 mouse myoblast cells were obtained from the National Center for Cell Science (NCCS), Pune, India and maintained in Dulbecco’s modified Eagle’s medium (DMEM) supplemented with 10% fetal bovine serum and 10 units/ml penicillin/streptomycin/glutamine. Cells were transfected with either scramble or miR-381-3p or miR-539-5p mimics (25 and 50 nM, Dharmacon, Lafayette Colorado, U.S.A.) and after 48 h, total RNA was isolated using TRIzol and 1 µg RNA was reverse transcribed and quantitative PCR was performed for *Nfkbia, Pik3r1* and *Cdkn2d* as described below.

### qRT-PCR validation

DEGs that mapped on to the most over-represented pathway were taken for validation in skeletal muscle tissues of animals of the same strain and age group and comparable body weight and blood glucose levels as mentioned above. A total of 1.5 µg of total RNA was used to synthesize cDNA using ReverseAid Reverse Transcriptase (Thermo Fisher Scientific, U.S.A.) with gene-specific primers (Supplementary Table S1). Quantitative PCR was performed for transcripts: *Pik3r1, Foxo1, Prkab2, Ppp1r3a, Srebf1, Pfkfb3* along with the lncRNAs: 8430426J06RiK, Foxo6os and 9830004L10RiK in a Step One Plus Real Time PCR (Applied Biosystems, Life Technologies, USA) using PowerUp SYBR Green Master Mix (Applied Biosystems, Life Technologies, USA). 18S ribosomal RNA (rRNA) was used as normalization control and the reaction specificity was verified by melt curve analysis. miRNAs altered in the skeletal muscles of db/db mice as reported in our previous study [29] were validated in the current set of animals. qRT-PCR for mature miRNAs was performed using 1 µg total RNA that was reverse transcribed and quantified using SYBR Green PCR Master Mix (Applied Biosystems, CA, U.S.A.) and miRNA-specific primers (Supplementary Table S1). U6 or Sno 234 was used as the normalization control. RNA from scramble or miR-381-3p or miR-539-5p transfected cells was reverse transcribed and transcript levels of *Nfkbia, Pik3r1* (miR-381-3p) and *Pik3r1, Cdkn2d* (miR-539-5p) were assessed using specific primers (Supplementary Table S1). 18S rRNA was used as normalization control. Relative transcript values were calculated by ΔΔ*C*_t_ method with *P*-value <0.05.

### Statistical analysis

Data were analyzed using two-tailed Student’s *t* test and values are represented as means ± S.E.M, *P*-value <0.05 is considered as statistically significant. Fisher’s exact test and Benjamini–Hochberg correction were used for RNA sequencing data analyses.

## Results

### RNA sequencing and data generation

RNA sequencing was performed with RNA isolated from skeletal muscle of 10-week-old male normal (C57BLKs- db/+) and diabetic (C57BLKs- db/db) mice (*n*=4). rRNA depleted RNA was used for strand-specific cDNA library preparation for transcriptome sequencing using the Illumina HiSeq 4000 platform. An average of 38.6 and 35.6 million raw reads were generated from 101-bp long paired-end sequencing from normal and diabetic samples, respectively (Supplementary Table S2). After removing adapter sequences and low-quality bases from the raw reads, the filtered and unique sequences were mapped on to the mouse reference genome (Gencode-M21 [65]) as described in the ‘Materials and methods’ section. Approx. 96.6 and 97.4% processed reads from the normal (db/+) and diabetic (db/db) samples respectively, mapped on to the mouse genome (Supplementary Table S2).

### Identification of DEGs

**Figure 1:**
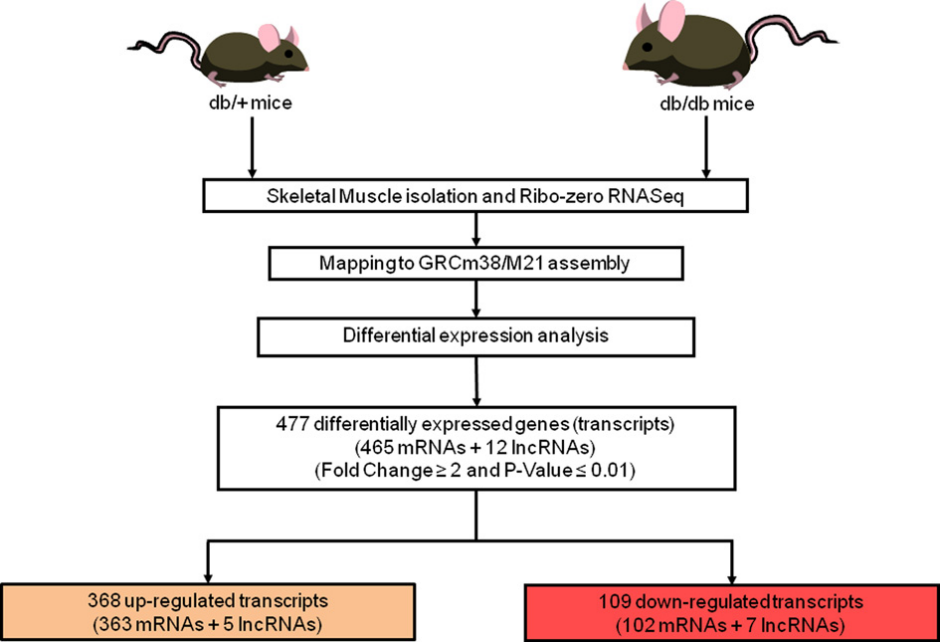
Schematic representation of the experimental work flow Overview of experimental and bioinformatics analysis pipeline of RNA-sequencing, to identify differentially regulated genes in skeletal muscles of db/db mice.

Gencode-M21 catalog was used for analyzing differential genes expression, annotation and evaluation. Expression of genes was assessed on the basis of their FPKM values. Cuffdiff [66] and DESeq 2.0 [67] tools were used for normalization of FPKM values and also for analyzing differential genes expression between normal and diabetic samples. All transcripts that were differentially altered in the skeletal muscle of db/db mice as compared with db/+ mice with a fold change ≥ ±2 and *P*-value <0.01 are listed as DEGs. Figure 1 summarizes the work flow and overview of the data analysis pipeline. Expression correlation among the genes expressed within the normal and diabetic groups is shown in Supplementary Figure S2.

**Figure 2:**
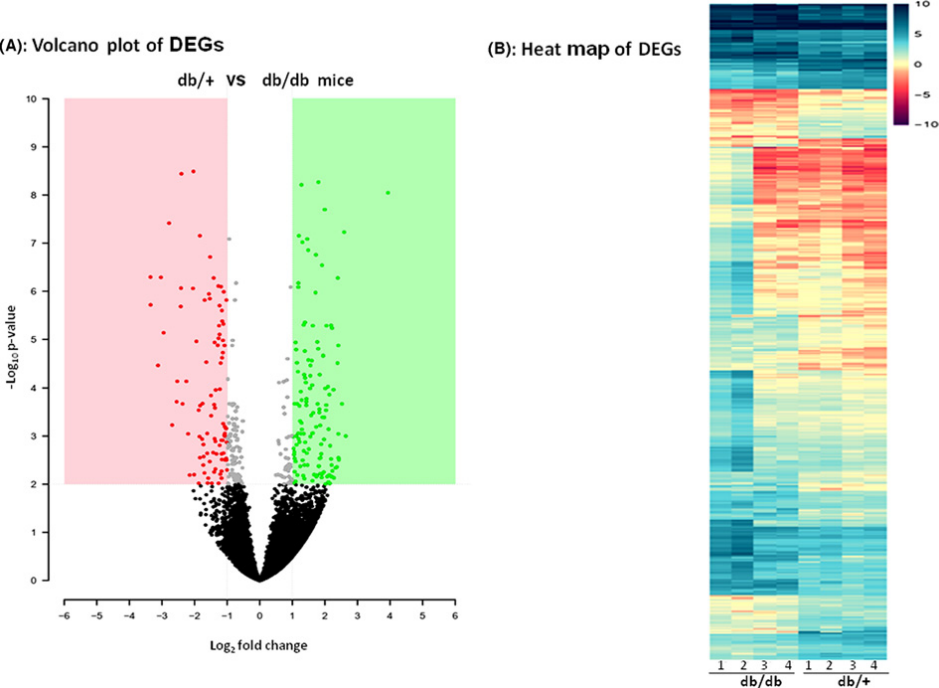
Representation of DEGs in diabetic (db/db) mice skeletal muscle (*n*=4) as compared with that in normal (db/+) mice (**A**) Volcano plot of DEGs. The dots in red represent the significantly (*P*<0.01) down-regulated genes and dots in green represent the significantly (*P*<0.01) up-regulated genes. (**B**) Heat map of DEGs. Figure represents the differences in log_2_ FPKM values of DEGs with fold change ≥ ±2.0 and *P*<0.01 between normal and diabetic animals.

As compared with normal animals, 465 mRNAs and 12 lncRNAs were differentially expressed in the skeletal muscle of diabetic animals with a fold change ≥ ±2.0 and *P*-value <0.01. Of these, 363 mRNAs and 5 lncRNAs were up-regulated and 102 mRNAs and 7 lncRNAs were down-regulated in diabetic animals (Supplementary Table S3A,B). The volcano plot of the altered genes in the diabetic mice skeletal muscle as compared with those in the normal animals along with the heatmap is shown in Figure 2A,B.

### Pathway enrichment analysis of DEGs

**Figure 3:**
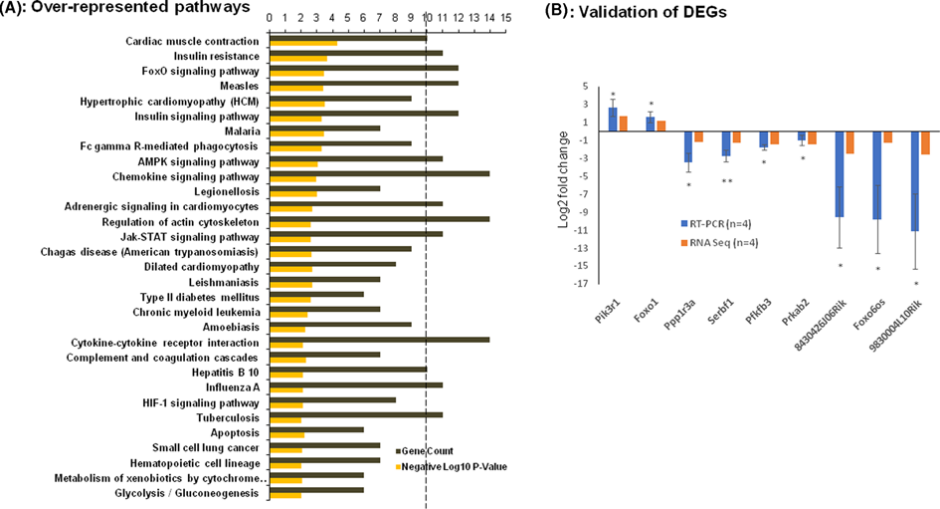
Pathway enrichment analysis of DEGs (**A**) Bar graph of significantly over-represented pathways that the differentially regulated genes map to along with number of genes in each pathway. The black dotted vertical line represents the minimum gene count with cutoff of 10 and yellow bars represent the negative log_10_*P*-value. (**B**) qRT-PCR validation of some differentially expressed mRNAs and lncRNAs as identified by RNA sequencing in the skeletal muscle of the db/db mice. Log_2_ fold changes with respect to normal animals is expressed (*n*=4). *P-value <0.05, **P-value <0.01.

To study the biological relevance and consequences of the DEGs in the skeletal muscle of diabetic mice, KEGG pathway enrichment was performed using the DAVID Bioinformatics Resources v6.8. Enrichment analysis with the differentially regulated 465 mRNAs and 12 lncRNAs showed 31 pathways to be majorly over-represented with an adjusted *P*-value <0.01 (Fischer’s exact test) (Figure 3A). Among these, insulin resistance, insulin signaling, FOXO signaling and AMPK signaling were major pathways to be over-represented with more than ten DEGs being involved in each pathway. On performing the pathway enrichment analyses with the up- and down-regulated transcripts independently, it was interesting to note that while chemokine and Foxo signaling were over-repesented among the up-regulated genes, pathways related to AMPK signaling, insulin signaling, insulin resistance and adipocytokine signaling were significantly over-represented within the down-regulated transcripts (Supplementary Figure S3A,B). All these pathways play important roles in the skeletal muscle to maintain glucose homeostasis. Impaired regulation of these pathways is believed to interfere with insulin signaling and lead to the diabetic phenotype. Interestingly, most transcripts namely, *Pck2, Prkab2, Pik3r1, Foxo1, Irs1* and *Pik3r5* are commonly involved in these pathways and this suggests a significant involvement and contribution of these genes in aberrant skeletal muscle metabolism during diabetes. The expression of some such common differentially regulated genes in these pathways along with a few lncRNAs was validated by qRT-PCR. As in RNA sequencing, while the levels of *Pik3r1* and *Foxo1* were up-regulated, that of *Ppp1r3a, Srebf1, Pfkfb3, Prkab2* and of the lncRNAs, 8430426J06Rik, Foxo6os and 9830004L10Rik were down-regulated in the diabetic mice skeletal muscle as compared with normal mice (Figure 3B).

**Figure 4:**
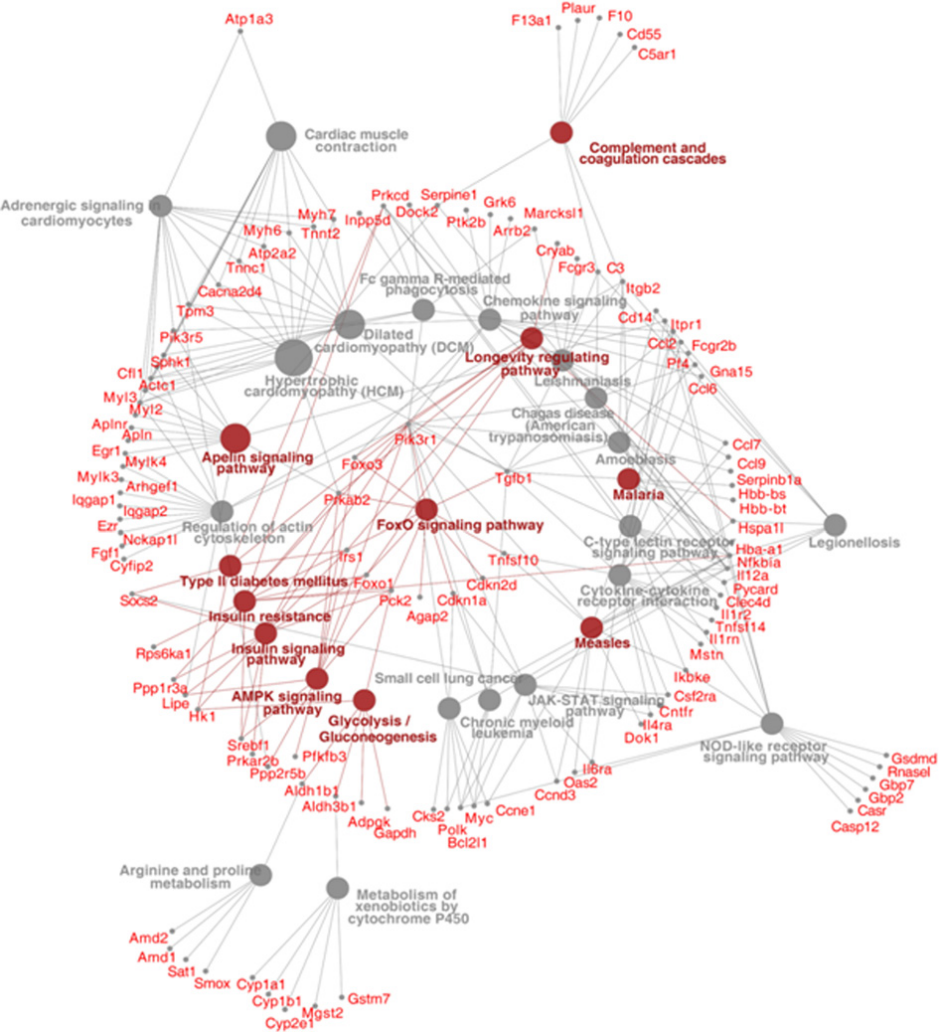
CytoScape and ClueGO derived correlation network among genes that mapped on to significantly enriched pathways DEG names are mentioned in red and significantly (*P*<0.05) over-represented pathways by these genes are denoted by brown circles. The grey circles represent pathways over-represented by the DEGs, but with a *P*-value >0.05.

To assess the potential cross-talk among these altered pathways, we constructed an interaction network between the differentially regulated genes and their implicated pathways using ClueGo [59] and Cytoscape [58] (Figure 4). This interaction network shows that the above-described pathways have significant interactions among each other and their aberrant regulation may affect the metabolism of other pathways. In this interaction network, most significant emergent pathways were those of apelin, insulin, FOXO, AMPK, T2DM and glycolysis/gluconeogenesis signaling, and deregulation of any of these pathways might promote irregular skeletal muscle metabolism and growth and this has been reported to be causative to several irregularities associated with the diabetic phenotype [68–70].

### Interaction networks among differentially regulated genes (DEGs)

**Figure 5:**
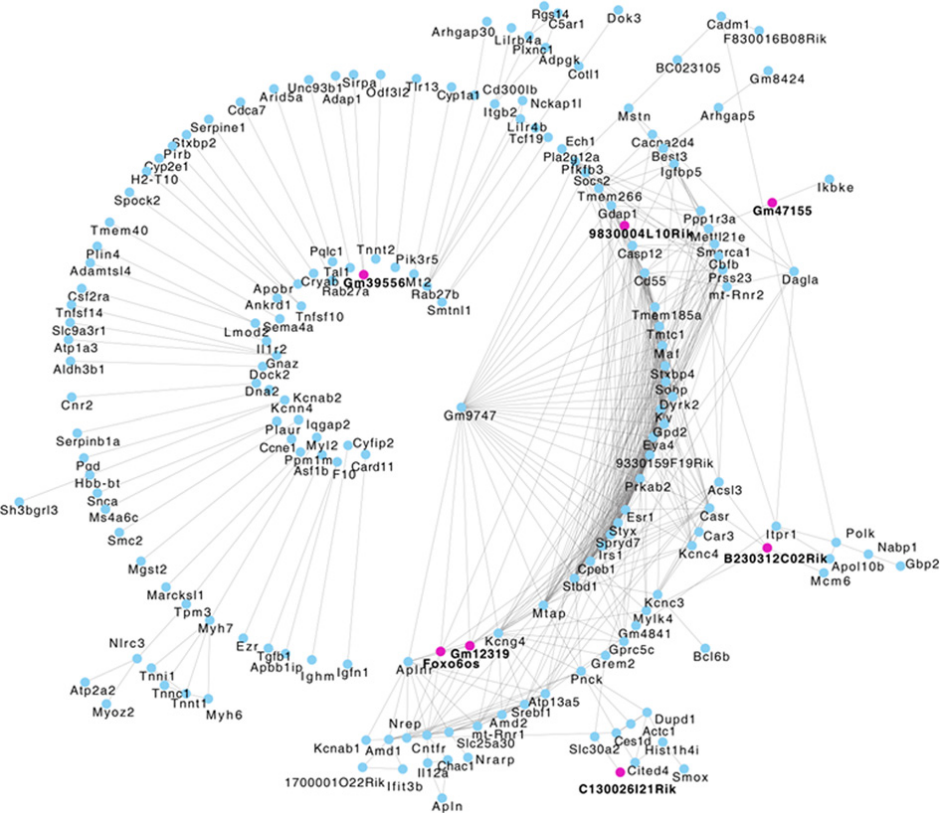
Correlation network among DEGs as derived by CytoScape Of the differentially expressed transcripts, 244 of them significantly (*P*<0.05) mapped in interaction networks and this included 7 dysregulated lncRNAs. Potential interactions between these 237 transcripts (cyan) and 7 lncRNAs (pink) is shown. The lncRNAs show direct and indirect interactions with other altered transcripts suggesting potential modes of lncRNA-mediated gene regulation.

To explore interactions among the DEGs, interaction networks were drawn using Cytoscape. Several differentially regulated genes depicted strong interactions with each other. Genes like, *Maf, Prkab2, Irs1, Pfkfb3, Socs2, Srebf1, Ppp1r3a, Pik3r5* and *Dyrk2*, showed multiple interactions with other altered genes. Prominently, *Prkab2, Irs1, Srebf1* and *Pfkfb3* that are common to several relevant pathways namely insulin signaling, AMPK signaling and FOXO signaling pathways, were majorly interactive (Figure 5). Of the 12 deregulated lncRNAs, 7 lncRNAs showed significant (*P*<0.05; Benjamini–Hochberg correction) interactions with other altered genes, specifically with the above-mentioned genes which suggest that these lncRNAs might have a regulatory role in their expression and/or metabolic function. All these suggest that the deregulated transcripts or differentially regulated genes in the skeletal muscle during diabetes cross-talk with each other and lncRNAs participate in these interactions and overall, these events might be critical in the altered skeletal muscle physiology during diabetes.

### Identification of potential miRNA–mRNA interactions

**Figure 6:**
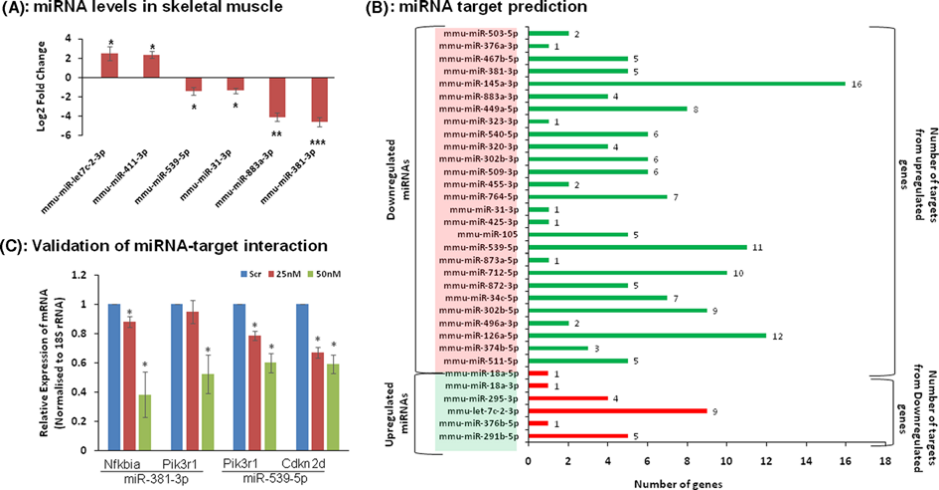
Prediction and validation of DEGs as potential targets of deregulated miRNAs in the db/db mice skeletal muscle (**A**) MiRNAs altered in the skeletal muscle of diabetic db/db mice were validated by qRT-PCR. One microgram of total RNA was reverse transcribed and quantified using by qRT-PCR and miRNA-specific primers. U6 or sno 234 was used as the normalization control. (**B**) Putative miRNA targets were extracted from miRDB and TargetScan, and targets of down-regulated miRNAs were matched to the up-regulated genes and *vice versa*. Green bars represent the number of up-regulated genes that are predicted to be targeted by down-regulated miRNAs and red bars represent the number of down-regulated mRNAs genes that are predicted to be targeted by up-regulated miRNAs. Numbers alongside the bars represent number of genes predicted as targets to the particular miRNA. (**C**) C2C12 cells were transfected with either the scramble (scr) or miR-381-3p or miR-539-5p (25-50 nM) and after 48 h, total RNA was isolated and the transcript levels of the predicted targets namely, *Nfkbia, Pik3r1* (miR-381-3p) and *Pik3r1, Cdkn2d* (miR-539-5p) were assessed by qRT-PCR using specific primers. 18S rRNA was used as normalization control. (A) *P<0.05, **P<0.01 and ***P<0.001 as compared with db/+ mice; (C) *P<0.05 as compared with scramble (scr).

In previous study from our laboratory, we had reported altered miRNA signatures in the skeletal muscle of diabetic db/db mice where 31 miRNAs were down-regulated and 10 were up-regulated [29]. Figure 6A shows RT-PCR validation of a few of these altered miRNAs in the skeletal muscles of the current set of db/db mice as compared with db/+ mice. Since miRNAs primarily act by binding to the 3′UTR of their target mRNAs thereby inhibiting translation or promoting mRNA degradation, we explored if any of the differentially regulated genes that are altered in the skeletal muscle during diabetes are predictably targeted by such altered miRNAs. Targets to the altered miRNAs were extracted using TargetScan and miRDB, and these were mapped on to the altered transcripts, as obtained in the present study. More specifically, predicted targets of the down-regulated miRNAs were mapped on the up-regulated transcripts and *vice versa*. Six up-regulated miRNAs had their predicted targets among the down-regulated transcripts and, likewise, predicted targets to 27 down-regulated miRNAs were among the up-regulated transcripts (Figure 6B). As shown in Figure 3A, relevant pathways like insulin signaling, FOXO signaling and AMPK signaling were significantly over-represented among the DEGs; we, therefore, explored the predicted interaction of the altered miRNAs with the genes relevant to these pathways, if any. Up-regulated genes like *Pik3r1, Foxo1, Hk1, Pik3r5, Tfgb1*, etc. were predicted to be targeted by the down-regulated miRNAs. Also, the down-regulated gene, *Pfkfb3* was predicted to be targeted by the up-regulated miRNAs, miR-291b-3p and miR-let7c-2-3p (Supplementary Table S4). We sought to validate some of these interactions *in-vitro* using C2C12 skeletal muscle cells. Overexpression of the miR-381-3p and miR-539-5p in C2C12 skeletal muscle cells led to an almost 500-fold increase in the endogenous levels of the respective miRNAs and this was associated with significant down-regulation of the predicted targets, namely *Nfkbia* and *Pik3r1* for miR-381-3p and *Pi3kr1* and *Cdknd2* for miR-539-5p (Figure 6C). Such inverse patterns of expression between the miRNAs and the corresponding predicted targets validate the negative regulation of the targets by miRNAs. This suggests that cellular levels of these transcripts might be determined by the altered miRNAs, consequently influencing the corresponding pathways and impairing skeletal muscle metabolism during diabetes.

## Discussion

In the present study, we aimed to construct a putative map of genetic interactions between the altered transcriptome and ncRNome in the skeletal muscle of mice during diabetes. Using RNA sequencing, we identified 465 mRNAs and 12 lncRNAs to be differentially expressed in the skeletal muscle during diabetes.

Using microarray and RNA sequencing, several studies have previously reported altered transcript signatures in the skeletal muscle during diabetes [71–76]. Consequent in-depth analyses of such studies have provided evidence that majority of the genome that is transcribed into RNA, also constitutes the transcription of ncRNAs that demonstrate varied cellular functions and regulation of various cellular processes. These indicate the presence of evident regulatory networks between transcripts and ncRNAs that are critical in diverse cellular processes.

Our study reveals that strong interacting networks exist between the altered mRNAs and lncRNAs in the skeletal muscle of diabetic db/db mice. Construction of lncRNA–mRNA networks interestingly, reveals potential cellular cross-talk between the corresponding lncRNA and mRNA. Here we show that, of the total mRNAs and lncRNAs altered, 7 lncRNAs and 237 altered mRNA transcripts are involved in a core co-expression interaction network, suggesting that these lncRNAs may alter and regulate the levels of these mRNA transcripts and therefore affect skeletal muscle metabolism and validation of these is worthy of further investigation. Two of the deregulated lncRNAs, namely, lncRNA Gm12319 and B230312C02Rik are reported to interact with the RNA-binding protein, Fus [77,78] and our current study identified this protein to be differentially regulated by a significant 1.6-fold in the skeletal muscle of db/db mice. Such reports substantiate the potential cellular existence of the above-derived lncRNA–mRNA interacting networks. The lncRNA, Foxo6os was found to be down-regulated in our study and such an expression pattern of this lncRNA during diabetes has also been reported in the recent study by Zhang et al., [28]. In the study, using RNA sequencing, the authors found 331 altered lncRNAs in the skeletal muscle of db/db mice. However, in our study, we found only 12 lncRNAs to be differentially regulated. This difference could be due to our use of a more stringent analysis pipeline using two tools, Deseq2 and Cuffdiff, and a more stringent *P*-value and FPKM cut-off value to identify DEGs. Yet, on comparing our data with that of Zhang et al. (2018) [28], we find 137 common mRNA transcripts to be the differentially regulated, in addition to the one common lncRNA as mentioned above. The small number of overlaps between the two studies is expected considering the varied *P*-values, analysis pipelines and sequencing criteria used in both the studies.

Functional enrichment analysis suggested that most of the DEGs are significantly over-represented and involved in pathways of insulin, FOXO and AMPK signaling. AMPK is a major energy sensor in the skeletal muscle and its activity here is regulated by the intensity and duration of exercise. It is important in glucose transport, mitochondrial biogenesis and insulin sensitivity in the skeletal muscle [79,80]. Obese and diabetic subjects have reduced stimulation of AMPK by exercise [81]. Among members of the FOXO1 family, overexpression of FOXO1 increases the percentage of fast twitch muscle fibers and decreases muscle size [82]. Inhibition of transcriptional activity of FOXO1 and FOXO3 increases skeletal muscle fiber area and myotube diameter in the soleus muscle [83,84]. Reports suggest that FOXO-driven protein degradation is responsible for muscle atrophy as seen during insulin-deficient diabetes [85]. These are indicative of the significance of these pathways in the skeletal muscle and their over-representation by the altered mRNA transcripts and lncRNAs suggests a possible critical role of these RNA species in overall skeletal muscle metabolism during diabetes.

In a previous study, we had reported an altered miRNA signature in the skeletal muscle of diabetic db/db mice [29]. We observed inverse patterns of expression between several deregulated miRNAs and their predicted targets that were among the differentially regulated transcripts (as obtained in the present study), all suggesting that such an altered miRNA signature might be responsible for the status of altered transcripts in the skeletal muscle of db/db mice. As a clue towards validation of these, our results show that overexpression of the miR-381-3p and miR-539-5p in C2C12 skeletal muscle cells significantly down-regulated the transcript levels of the predicted targets, namely *Nfkbia* and *Pik3r1* (for miR-381-3p) and *Pi3kr1* and *Cdknd2* (for miR-539-5p). It is reported that miR-18a, one of the altered miRNAs, directly regulates the expression of Estrogen Receptor-1 (ESR-1) [86] and miR-34c targets Sema-4b [87]. High-throughput sequencing techniques like HITS-CLIP have provided evidence towards validations of other miRNA–mRNA target pairs. miR-712-5p has been shown to target F10 and EIF4 [88–90]; miR-449a targets Sema4b [88] and miR-381-3p targets Npr3 and Pik3r1 [89–91]. All these substantiate and provide support towards the validation of our miRNA–mRNA predictions. Similar correlations between the miRNome and transcriptome have been reported previously [30,92–95] in diverse pathological states and such studies offer profound understanding of regulatory networks that underlie aberrant cellular physiology under these conditions.

To conclude, the present study puts forth an in-depth analysis and comprehensive understanding of the cross-talk and interaction between the deregulated ncRNome (altered miRNAs and lncRNAs) and transcriptome (altered mRNA transcripts) in the skeletal muscle of diabetic db/db mice. Such biological regulatory networks enable identification of appropriate functional and complex events within a cell and therefore, for a metabolic and multifactorial disease as complex as diabetes, this is critical in identifying the underlying mechanisms to enable a better understanding of the disease pathology. However, further validations *in-vitro* and in other animal models of diabetes together with assessment in humans would be valuable in establishing the conclusions presented. Yet, the study might be exploited as a possible prerequisite for potential therapeutic interventions and identification of targets to combat this metabolic disease.

## Supplementary Material

Supplementary Figures S1-S3 and Tables S1-S4

## Data Availability

The data that support the findings of the present study are openly available in the NCBI SRA database (Sequence Read Archive, https://submit.ncbi.nlm.nih.gov/) with the accession number: SRP238785.

## Abbreviations

AMPK: AMP-activated protein kinase
DEG: differentially expressed gene
FPKM: fragments per kilobase of transcripts per million mapped reads
lncRNA: long non-coding RNA
miRNA: microRNA
ncRNA: non-coding RNA
rRNA: ribosomal RNA
T2DM: Type 2 diabetes mellitus
