# Supplementary material for: RNA sequencing reveals potential interacting networks between the altered transcriptome and ncRNome in the skeletal muscle of diabetic mice
Source: Biosci Rep. 2021 Jul 12;41(7):BSR20210495. doi: 10.1042/BSR20210495 (PMC8276098; doi:10.1042/BSR20210495)
Supplement: Supplementary Figures S1-S3 and Tables S1-S4 [file BSR-2021-0495_supp.pdf]

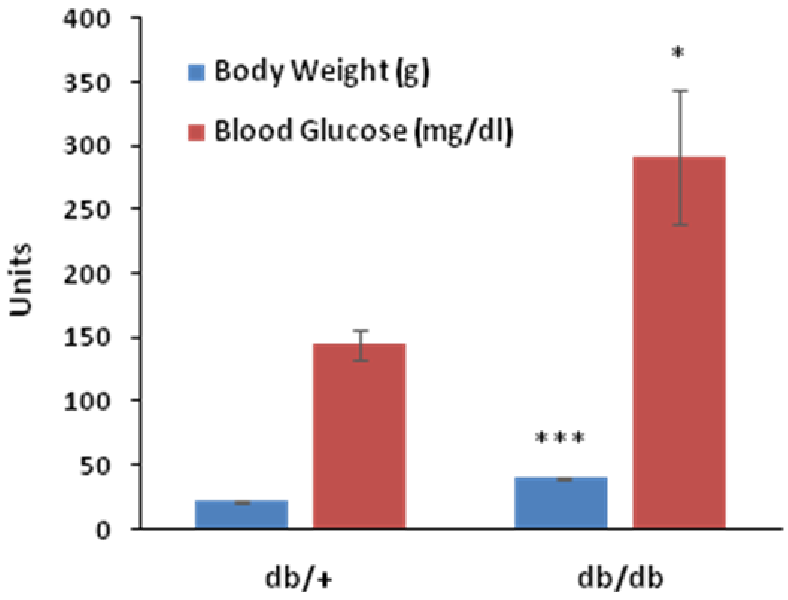

Supplementary Figure 1: Body weight and glucose levels of normal (db/+) and diabetic (db/db) animals taken for the study.

*p<0.05 and ***p<0.001 as compared to db/+

(a) Correlation in normal (db/+) mice

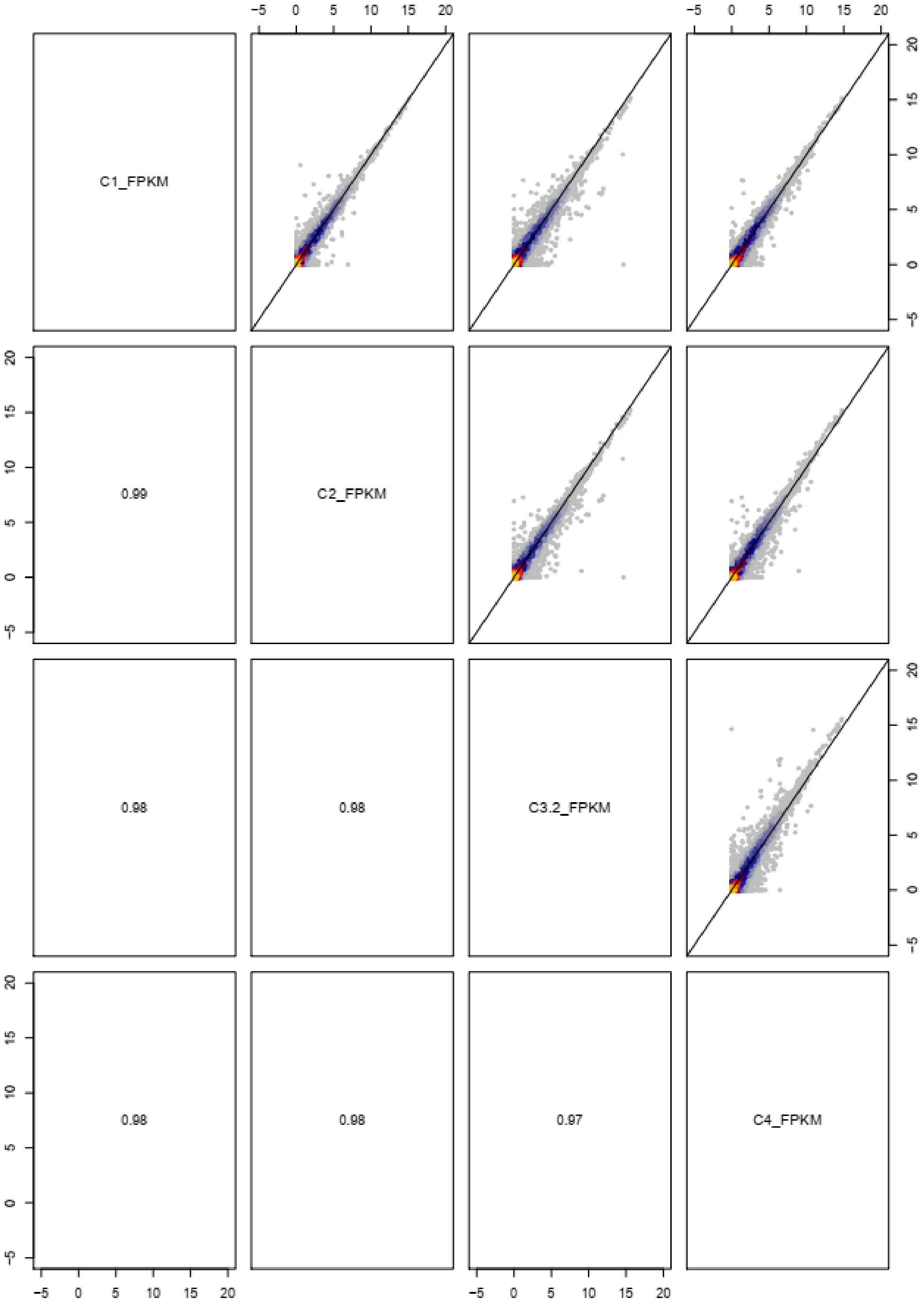

(b) Correlation in diabetic (db/db) mice

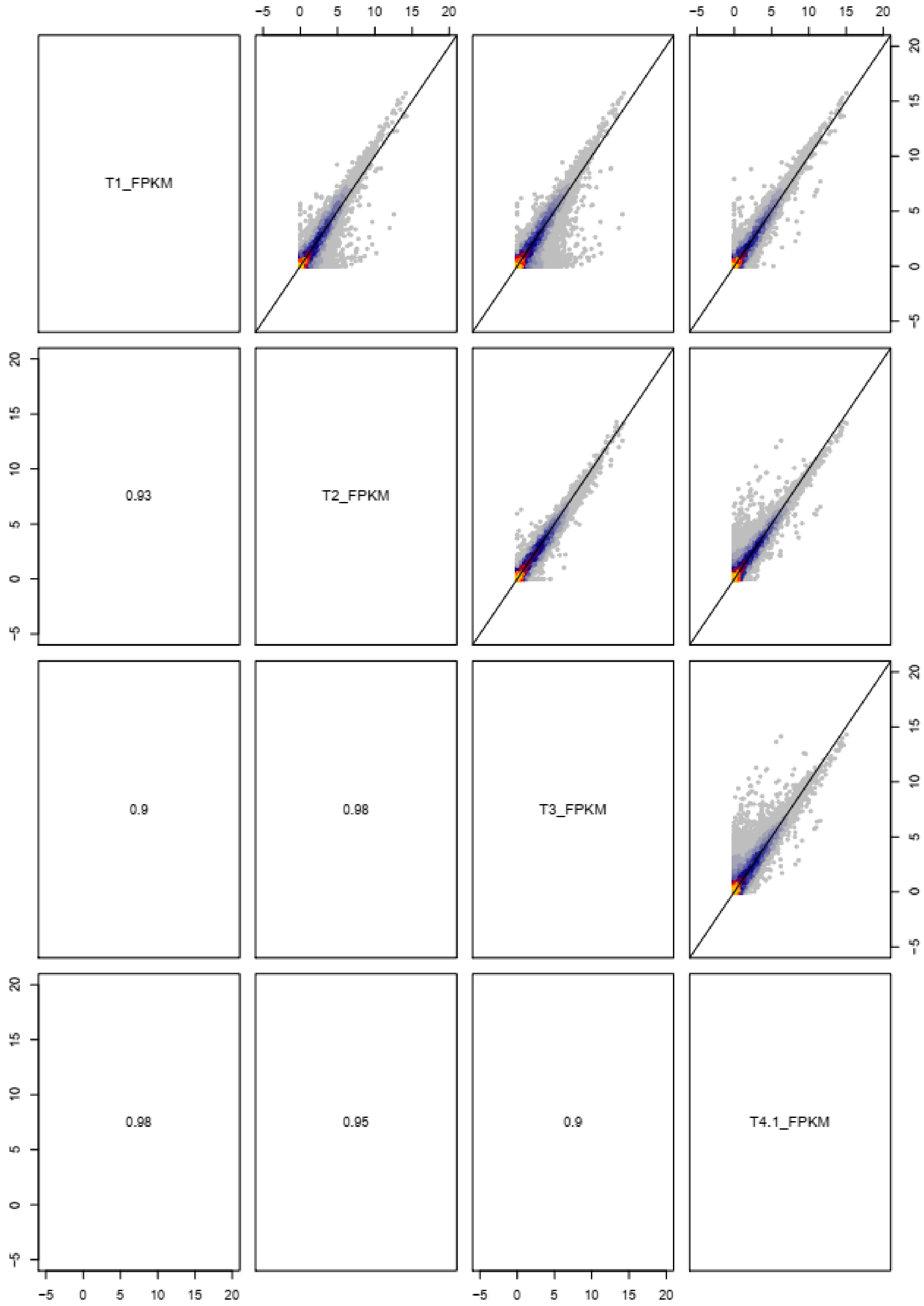

Supplementary Figure 2: Correlation among FPKM values of normal (a) and diabetic (b) mice used for RNA sequencing.

a. Over-represented pathways among up-regulated genes

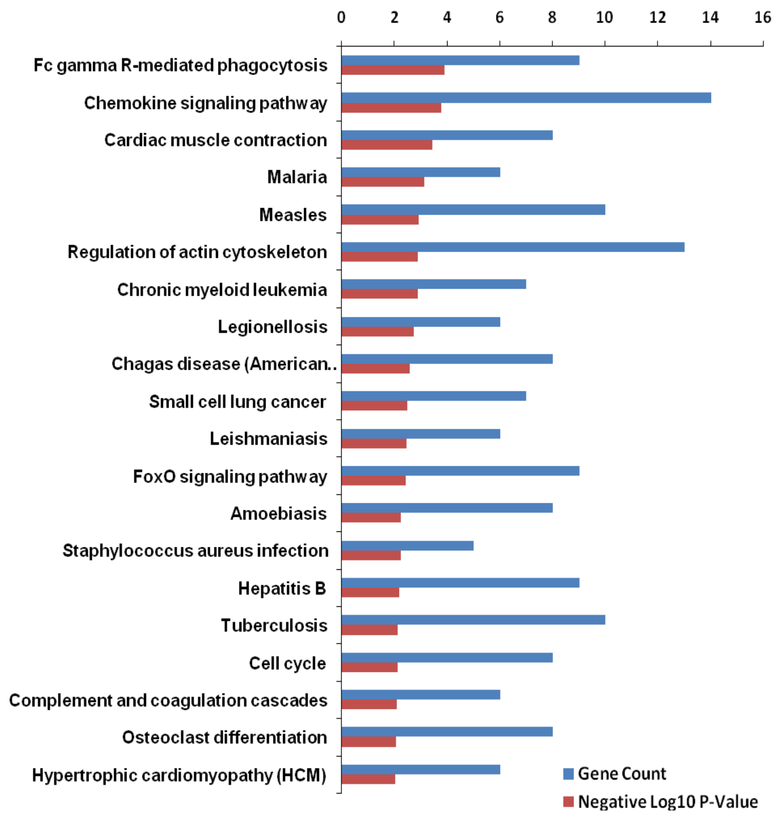

b. Over-represented pathways among down-regulated genes

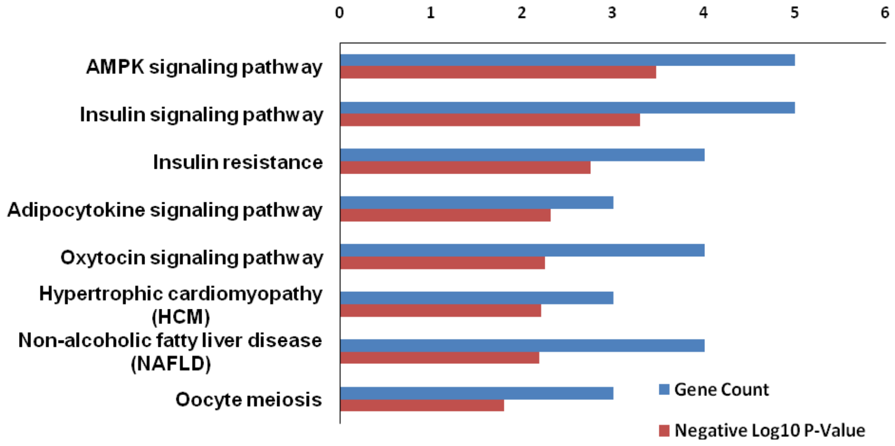

Supplementary Figure 3: Pathway enrichment analysis of differentially expressed genes

SUPPLEMENTARY INFORMATION

Supplementary Table 1: Primer sequences used for qRT-PCR validation

Gene Name	Primer Sequence
Pik3r1 FP	5’-CTATGCCTGCTCCGTAGTGG-3’
Pik3r1 RP	5’-CTTCTCAGAGGCCTTGGGTG-3
Foxo1 FP	5′-ATTCGGTCATGCCAGCGTAT-3′
Foxo1 RP	5′-TGTGTGAGGCATGGTGTTCA-3
Ppp1r3a FP	5’-TCCCGGAGATTTCCTTTGC-3’
Ppp1r3a RP	5’-CGGCTTTCTGGACTTGGAGT-3’
Prkab2 FP	5’-TCACCTACCTCCTGGCTCTC-3’
Prkab2 RP	5’-AGAGTCTCAGCTGAGGGAGG-3’
Pfkfb3 FP	5’-GTCGCCGAATACAGCTACGA-3’
Pfkfb3 RP	5’-GAGCCCCACCATCACAATCA-3,
Srebf1 FP	5’-TAGTGACTCTGAGCCCGACA-3’
Srebf1 RP	5’-ATCTCTGCTCTCTGCCTCCA-3’
8430426J06Rik FP	5’-TGTAGGGAGCAGTTTGGCAG-3’
8430426J06Rik RP	5’-GTCTGGGCTTTTGGTTCCCT-3’
Foxo6os FP	5’-GTCTCCTGGGTTTCCTGTGG-3’
Foxo6os RP	5’-CTCCCCACATCCTCAAACCC-3’
9830004L10Rik FP	5’-ACATGCAGATGCTGGGACAA-3’
9830004L10Rik RP	5’-AGCAAAGGTGGGTCAGGAAC-3’
Pik3r1 FP	5’-CTATGCCTGCTCCGTAGTGG-3’
Pik3r1 RP	5’-CTTCTCAGAGGCCTTGGGTG-3’
Nfkbia FP	5’-GAGACCTGGCCTTCCTCAAC-3’
Nfkbia RP	5’-CAAGACTGCTACACTGGCCA-3’
Cdkn2d FP	5’-CAGCCCTGACCTTAACTGGG-3’
Cdkn2d RP	5’-GTCTTGCCAAAGCGGTTCAG-3’
miR-539-5p SLP	5’-CTCAACTGGTGTCGTGGAGTCGGCAATTCAGTTGAGACACACCA-3’
miR-539-5p FP	5’-ACACTCCAGCTGGGGGAGAAATTATCCTTG-3’
miR-381-3p SLP	5’-CTCAACTGGTGTCGTGGAGTCGGCAATTCAGTTGAGACAGAGAG-3’
miR-381-3p FP	5’-ACACTCCAGCTGGGTATACAAGGGCAAGCT-3’
miR-31-3p SLP	5’-CTCAACTGGTGTCGTGGAGTCGGCAATTCAGTTGAGGATGGCAA-3’
miR-31-3p FP	5’- ACACTCCAGCTGGGTGCTATGCCAACATATT-3’

miR-883-3p SLP	5’-CTCAACTGGTGTCGTGGAGTCGGCAATTCAGTTGAGATACTGAG-3’
miR-883-3p FP	5’- ACACTCCAGCTGGGTAACTGCAACATCTCT-3’
miR-universal RP	5’-GTGTCGTGGAGTCGGCAATTC-3’
Sno234 SLP	5’-GGATCGCCTCTCAGTGGTAG-3’
Sno234 FP	5’-GGCTTTTGGAATGAATCTAAGT-3’
Sno234 RP	5’-GAGGTATTCGCACCAGAGGA-3’
miR-let7c-2 FP	5’- TGAGGTAGTAGGTTGTATGGTT-3’
miR-411 FP	5’- TAGTAGACCGTATAGCGTACG-3’
U6 FP	5’-CGCTTCGGCAGCACATATAC-3’
Universal-RP for miR-411, let-7c, U6	5'-CTCAATCGTACATAGAAACAGGGATC-3'
18s rRNA FP	5’-GTAACCCGTTGAACCCCATT-3’
18s rRNA RP	5’-CCATCCAATCGGTAGTAGCG-3’

FP: Forward primer; RP: Reverse primer; SLP: Stem loop primer

<u>Supplementary Table 2</u>: Data summary of RNA sequencing in normal (db/+) and diabetic (db/db) mice

**Sample**	**Raw Reads (x2)**	**Trimmed Reads (x2)**	**% Retained**	**% Discarded**	**% Alignment**
C1	20219922	19726700	97.5607126	2.439287	97.7559855
C2	20938403	20431035	97.5768544	2.423146	97.6344566
C3	15772588	15445433	97.9258001	2.0742	93.5299127
C4	20614449	20151238	97.752979	2.247021	97.5515723
**Normal mice (db/+) (Average)**	**19386340**	**18938601**	**97.70408653**	**2.2959135**	**96.61798178**
T1	16161140	15679238	97.0181435	2.981856	97.3585036
T2	19256753	18791677	97.584868	2.415132	97.6374009
T3	18451581	17994780	97.524326	2.475674	97.8634165
T4	17672104	17311527	97.9596261	2.040374	96.6543853
**Diabetic mice (db/db) (Average)**	**17885394**	**17444305**	**97.5217409**	**2.478259**	**97.37842658**

<u>Supplementary Table 3</u>: Differentially expressed transcripts in skeletal muscles of db/db mice (Fold change ≥±2.0 and p <0.01)

A. Differentially expressed lncRNAs

Ensemble Gene ID	Gene Symbol	Control FPKM	Diabetic FPKM	Log2 FC	p--value
ENSMUSG00000116380.1	Gm39556	0.1382329	1.3636978	1.58994	0.008522
ENSMUSG00000099906.2	Gm28653	0.1738916	1.2351885	2.016742	0.000247
ENSMUSG00000097247.2	1500012K07Rik	0.949887	6.4217525	2.161238	2.30E-05
ENSMUSG00000066170.7	E230001N04Rik	0.1880553	0.752311	1.883306	0.000976
ENSMUSG00000073535.5	Gm5532	1.1244888	4.16768	1.666042	0.000131
ENSMUSG00000084929.1	Foxo6os	7.49939	3.343935	-1.22203	7.34E-07
ENSMUSG00000084843.1	B230312C02Rik	4.86994	2.1699375	-1.04863	0.003242
ENSMUSG00000114019.1	Gm47155	5.7112675	2.4882625	-1.00198	0.00162
ENSMUSG00000087523.1	Gm12319	132.88725	54.807425	-1.23274	3.12E-08
ENSMUSG00000115970.1	8430426J06Rik	3.6215575	0.6649498	-1.81181	0.000279
ENSMUSG00000099552.1	9830004L10Rik	1.2042975	0.21796	-2.36791	1.73E-06
ENSMUSG00000092397.7	C130080G10Rik	5.7370575	0.501349	-3.35217	8.45E-10

B. Differentially expressed mRNAs

Ensemble Gene ID	Gene Symbol	Control FPKM	Diabetic FPKM	Log2 FC	p-value
ENSMUSG00000032315.6	Cyp1a1	1.0804153	13.92966	3.53128	5.60E-32
ENSMUSG00000031762.7	Mt2	13.231975	166.8615	3.43037	4.59E-31
ENSMUSG00000033826.10	Dnah8	0.037706	3.169749	2.64004	1.25E-05
ENSMUSG00000021062.15	Rab15	0.5198133	4.349793	2.59075	5.74E-11
ENSMUSG00000061878.15	Sphk1	0.3848332	2.507869	2.52355	1.70E-06
ENSMUSG00000025473.16	Adam8	0.5918335	7.529643	2.4124	1.08E-05
ENSMUSG00000020908.14	Myh3	0.555531	4.363873	2.4119	6.33E-08
ENSMUSG00000050335.17	Lgals3	9.464435	65.03333	2.39426	9.93E-10
ENSMUSG00000042045.6	Sln	5.2734525	43.5811	2.38579	1.71E-07
ENSMUSG00000031765.8	Mt1	47.121375	252.2885	2.35009	1.26E-28
ENSMUSG00000021322.8	Aoah	0.1049465	1.543372	2.32843	8.78E-05
ENSMUSG00000011305.11	Plin5	8.8318675	46.64228	2.29327	6.31E-17
ENSMUSG00000031722.10	Hp	19.045728	203.2481	2.22754	0.0001
ENSMUSG00000053964.17	Lgals4	2.0955165	10.18567	2.21907	2.23E-08
ENSMUSG00000042349.13	Ikbke	0.1931633	1.723563	2.20454	7.40E-05

ENSMUSG00000073730.2	4933415F23Rik	0.2493445	2.367495	2.191	0.00015
ENSMUSG00000033685.13	Ucp2	14.720885	76.12245	2.19003	1.82E-08
ENSMUSG00000024660.9	Incenp	1.87776	11.19634	2.18922	5.08E-05
ENSMUSG00000035373.2	Ccl7	0.3378478	3.180223	2.15082	0.00024
ENSMUSG00000026073.13	Il1r2	0.9651983	9.703681	2.13733	0.00024
ENSMUSG00000042759.12	Apobr	0.5235813	4.036921	2.13275	0.00013
ENSMUSG00000029816.10	Gpnmb	0.5783718	3.079538	2.11113	7.87E-06
ENSMUSG00000052477.15	C130026I21Rik	0.2054301	1.351146	2.07704	0.00054
ENSMUSG00000004626.14	Stxbp2	3.5175175	16.29125	2.06936	0.00015
ENSMUSG00000040752.16	Myh6	3.445065	16.19515	2.04601	1.94E-08
ENSMUSG00000021091.8	Serpina3n	1.453158	7.41231	2.03429	2.61E-06
ENSMUSG00000002257.8	Def6	0.9603443	5.996937	2.01932	0.00035
ENSMUSG00000053093.16	Myh7	69.66315	378.903	2.01063	7.01E-07
ENSMUSG00000062248.5	Cks2	2.015237	17.58332	2.00378	0.00077
ENSMUSG00000020108.4	Ddit4	9.0082775	38.80458	1.99461	1.67E-11
ENSMUSG00000028860.13	Sytl1	0.2120075	0.933007	1.98411	0.00087
ENSMUSG00000064179.13	Tnnt1	130.23405	637.5053	1.97932	3.99E-06
ENSMUSG00000054342.8	Kcnn4	0.646417	4.646612	1.97868	0.00044
ENSMUSG00000072082.7	Ccnf	0.3176198	2.294206	1.96744	0.00041
ENSMUSG00000013155.10	Enkd1	0.4298815	2.373499	1.96427	0.00011
ENSMUSG00000024640.9	Psat1	0.9122935	5.255048	1.94552	1.09E-07
ENSMUSG00000030470.15	Csrp3	120.76935	563.7748	1.92727	3.94E-06
ENSMUSG00000021123.12	Rdh12	0.5588523	3.693952	1.9236	0.00083
ENSMUSG00000032020.15	Ubash3b	0.1393689	0.96897	1.92233	0.00079
ENSMUSG00000020143.15	Dock2	0.2524465	2.164714	1.92138	0.00067
ENSMUSG00000020340.16	Cyfip2	0.7964235	4.35785	1.91621	0.00012
ENSMUSG00000039236.18	Isg20	2.12217	12.74312	1.91489	0.00055
ENSMUSG00000037139.15	Myom3	10.481233	46.35968	1.91081	4.59E-10
ENSMUSG00000029205.12	Chrna9	0.4092323	2.284418	1.89963	0.00016
ENSMUSG00000059900.14	Tmem40	1.0023973	7.891664	1.89755	0.00145
ENSMUSG00000028931.11	Kcnab2	0.5365555	3.850154	1.8966	0.00086
ENSMUSG00000030144.4	Clec4d	0.5071945	5.090854	1.8889	0.00166
ENSMUSG00000036564.17	Ndrg4	1.2126953	4.537725	1.88755	3.72E-05
ENSMUSG00000026418.16	Tnni1	86.312425	427.8505	1.88574	2.10E-05
ENSMUSG00000023169.15	Slc38a1	0.1221282	0.856432	1.8806	0.0016
ENSMUSG00000043091.9	Tuba1c	3.0318275	14.09906	1.86492	4.67E-05
ENSMUSG00000022436.16	Sh3bp1	0.9600868	4.078559	1.86031	0.00044
ENSMUSG00000037337.11	Map4k1	0.4147873	3.469911	1.8593	0.00128
ENSMUSG00000058818.13	Pirb	0.885648	7.269865	1.85741	0.00173

ENSMUSG00000061877.13	BC048679	6.7611125	35.89463	1.85518	6.32E-06
ENSMUSG00000036768.6	Kif15	0.6997213	3.63572	1.85372	0.00197
ENSMUSG00000073940.3	Hbb-bt	37.539	329.0091	1.8534	0.0018
ENSMUSG00000024885.9	Aldh3b1	1.3371915	8.377493	1.84938	0.00084
ENSMUSG00000052760.16	A630001G21Rik	0.3773955	2.100866	1.84451	0.00142
ENSMUSG00000058126.7	Tpm3-rs7	7.300915	33.08933	1.84069	4.85E-05
ENSMUSG00000005470.8	Asf1b	0.9020433	6.312167	1.84007	0.00159
ENSMUSG00000069919.7	Hba-a1	347.743	2110.311	1.83674	0.00094
ENSMUSG00000023267.10	Gabrr2	1.0772663	4.219705	1.83055	2.46E-06
ENSMUSG00000018774.13	Cd68	2.6128675	10.20118	1.8285	3.00E-06
ENSMUSG00000022945.12	Chaf1b	0.1473421	1.309495	1.8139	0.00203
ENSMUSG00000031698.14	Mylk3	0.1035268	0.932418	1.81359	0.00071
ENSMUSG00000024803.9	Ankrd1	3.2301575	14.07766	1.81314	7.98E-05
ENSMUSG00000091898.8	Tnnc1	212.59315	986.6305	1.81277	7.81E-05
ENSMUSG00000032690.16	Oas2	0.356923	2.237431	1.80646	0.00097
ENSMUSG00000030793.4	Pycard	2.041145	11.56305	1.80255	0.00109
ENSMUSG00000040740.7	Slc25a34	4.5248325	15.98	1.80045	3.38E-12
ENSMUSG00000025172.3	Ankrd2	122.72255	467.3025	1.79521	7.50E-08
ENSMUSG00000062585.11	Cnr2	0.0904705	0.823722	1.78991	0.00296
ENSMUSG00000038775.14	Vill	0.5125888	3.560154	1.78843	0.00167
ENSMUSG00000033508.7	Asprv1	0.5273558	9.277104	1.78681	0.0031
ENSMUSG00000000320.10	Alox12	0.6197303	3.728765	1.77872	0.00027
ENSMUSG00000039109.16	F13a1	2.698035	12.75091	1.77749	0.00028
ENSMUSG00000049130.6	C5ar1	0.883288	5.564774	1.77739	0.00167
ENSMUSG00000030278.11	Cidec	5.3247425	21.15757	1.77551	1.75E-06
ENSMUSG00000037661.14	Gpr160	0.5433472	2.63003	1.77511	0.00162
ENSMUSG00000072949.6	Acot1	1.33071	6.414978	1.76867	0.00066
ENSMUSG00000035963.8	Odf3l2	7.8063425	30.7286	1.76656	4.85E-08
ENSMUSG00000074604.9	Mgst2	1.4321075	13.35189	1.76396	0.0028
ENSMUSG00000038963.15	Slco4a1	0.0815676	0.807611	1.75882	0.00343
ENSMUSG00000035385.5	Ccl2	0.446634	4.392095	1.75718	0.00302
ENSMUSG00000027907.4	S100a11	51.4484	225.4708	1.7523	0.00015
ENSMUSG00000036672.5	Cenpt	0.6671138	1.935215	1.74692	0.00072
ENSMUSG00000037280.12	Galnt6	0.0874219	0.750292	1.74406	0.00283
ENSMUSG00000013936.12	Myl2	326.41055	1394.487	1.74216	0.00014
ENSMUSG00000044468.14	Tent5c	0.66412	3.304547	1.74186	0.00156
ENSMUSG00000028843.8	Sh3bgrl3	30.173525	121.0258	1.74025	0.00015
ENSMUSG00000059326.7	Csf2ra	2.1184425	15.40836	1.73882	0.00366
ENSMUSG00000026832.12	Cytip	0.3352669	1.970476	1.73627	0.00244

ENSMUSG00000052085.7	Dock8	0.3726718	2.464174	1.73348	0.00161
ENSMUSG00000040485.5	Lrrc52	1.8133125	6.45324	1.73142	6.92E-06
ENSMUSG00000002068.16	Ccne1	0.549127	2.891869	1.72982	0.00236
ENSMUSG00000030263.13	Lrmp	0.980243	6.356421	1.7271	0.00243
ENSMUSG00000044447.5	Dock5	0.2531478	1.502045	1.71469	0.00248
ENSMUSG00000028773.8	Fabp3	694.3395	2447.93	1.71198	2.73E-09
ENSMUSG00000046694.6	Tent5b	0.3038688	0.94928	1.71094	0.00061
ENSMUSG00000052087.14	Rgs14	0.436595	3.099871	1.70819	0.0043
ENSMUSG00000001865.2	Cpa3	0.6136915	2.951235	1.7065	0.00039
ENSMUSG00000050410.15	Tcf19	0.5763323	4.08302	1.70618	0.00236
ENSMUSG00000026355.11	Mcm6	1.4405578	8.791643	1.70428	0.0037
ENSMUSG00000044734.16	Serpinb1a	4.6347	32.80517	1.70382	0.00385
ENSMUSG00000063193.8	Cd300lb	0.2984413	2.035047	1.70282	0.00361
ENSMUSG00000112023.1	Lilr4b	0.8124063	7.386199	1.69538	0.00496
ENSMUSG00000025889.13	Snca	2.3079425	13.44603	1.69294	0.00264
ENSMUSG00000036875.15	Dna2	0.3494195	1.78367	1.69134	0.00351
ENSMUSG00000040907.15	Atp1a3	0.5058315	2.472287	1.68832	0.00199
ENSMUSG00000031444.16	F10	0.5324783	4.137733	1.67891	0.00502
ENSMUSG00000005824.7	Tnfsf14	0.1679478	1.488459	1.67368	0.00541
ENSMUSG00000052305.6	Hbb-bs	412.5475	2282.748	1.67307	0.00313
ENSMUSG00000056413.16	Adap1	0.9116618	4.345293	1.66795	0.00177
ENSMUSG00000033777.4	Tlr13	0.1761834	1.441493	1.66448	0.00491
ENSMUSG00000046223.10	Plaur	1.6528288	8.896446	1.66347	0.00344
ENSMUSG00000059498.13	Fcgr3	3.6470725	17.05465	1.65786	0.0022
ENSMUSG00000005233.16	Spc25	0.6504275	4.172064	1.65621	0.00593
ENSMUSG00000053398.11	Phgdh	1.0941395	4.635848	1.65615	0.00048
ENSMUSG00000029373.7	Pf4	14.308795	84.50885	1.65311	0.00389
ENSMUSG00000001750.16	Tcirg1	3.989855	14.43936	1.6519	0.00031
ENSMUSG00000041859.10	Mcm3	3.97898	16.98289	1.64131	0.00184
ENSMUSG00000040675.17	Mthfd1l	0.1812917	1.363292	1.64099	0.00522
ENSMUSG00000029223.12	Uchl1	3.414695	11.84168	1.6407	3.08E-06
ENSMUSG00000037572.17	Wdhd1	0.2309653	1.885423	1.64028	0.00565
ENSMUSG00000020684.14	Rasl10b	0.1456481	0.87968	1.63124	0.00189
ENSMUSG00000027035.10	Cers6	0.2108766	1.031155	1.62998	0.00396
ENSMUSG00000000552.10	Zfp385a	3.936815	13.41192	1.62837	1.94E-08
ENSMUSG00000037095.8	Lrg1	26.4661	93.54748	1.62736	9.86E-06
ENSMUSG00000005339.10	Fcer1a	0.2060766	1.50102	1.62341	0.00688
ENSMUSG00000024511.15	Rab27b	0.122736	0.961476	1.62277	0.00651
ENSMUSG00000038147.13	Cd84	0.438326	3.030923	1.62158	0.00435

ENSMUSG00000020901.13	Pik3r5	0.39831	1.583822	1.62121	0.00475
ENSMUSG00000002910.11	Arrdc2	1.1918913	4.754405	1.61827	0.0002
ENSMUSG00000040312.14	Cchcr1	0.240101	1.059731	1.61639	0.00271
ENSMUSG00000035232.8	Pdk3	0.6387175	2.526073	1.61402	0.00322
ENSMUSG00000002997.15	Prkar2b	1.2551373	5.71699	1.614	0.00131
ENSMUSG00000031377.11	Bmx	0.2236648	0.971299	1.61398	0.00329
ENSMUSG00000027861.13	Casq2	13.392393	43.55163	1.609	5.82E-07
ENSMUSG00000027134.4	Lpcat4	1.42553	5.487635	1.60739	0.0021
ENSMUSG00000026981.15	Il1rn	0.482181	2.255416	1.60426	0.00483
ENSMUSG00000048865.16	Arhgap30	0.456221	3.174685	1.59953	0.00777
ENSMUSG00000112148.1	Lilrb4a	1.154015	8.977501	1.59915	0.00808
ENSMUSG00000020838.12	Slc6a4	0.1593515	1.226725	1.59876	0.00769
ENSMUSG00000026786.14	Apbb1ip	0.6739168	3.865293	1.59743	0.00402
ENSMUSG00000006221.7	Hspb7	166.44575	541.022	1.59456	1.44E-07
ENSMUSG00000033589.4	Reep4	1.4842175	4.886968	1.58547	0.00097
ENSMUSG00000079419.4	Ms4a6c	1.3983115	7.398066	1.58394	0.00635
ENSMUSG00000021676.9	Iqgap2	0.2555595	1.234051	1.58147	0.00692
ENSMUSG00000032202.11	Rab27a	1.3043378	5.7045	1.57688	0.00332
ENSMUSG00000026414.13	Tnnt2	1.544506	6.253593	1.57684	3.53E-06
ENSMUSG00000028961.15	Pgd	10.516468	36.29855	1.57606	0.00048
ENSMUSG00000028064.17	Sema4a	1.955935	9.61126	1.57457	0.00801
ENSMUSG00000027068.6	Dhrs9	0.0865933	0.680191	1.57343	0.00922
ENSMUSG00000053158.10	Fes	2.149965	10.99538	1.57335	0.00711
ENSMUSG00000035711.5	Dok3	1.7755325	14.08411	1.57268	0.00886
ENSMUSG00000022488.9	Nckap1l	1.1154955	5.931879	1.57091	0.00822
ENSMUSG00000026274.11	Pask	0.0754813	0.770556	1.56706	0.00788
ENSMUSG00000022099.17	Dmtn	0.8642305	3.623664	1.56645	0.00336
ENSMUSG00000074785.5	Plxnc1	0.1402972	0.815946	1.56208	0.00595
ENSMUSG00000026288.14	Inpp5d	0.9674435	3.363098	1.56159	0.00253
ENSMUSG00000034792.8	Gna15	0.4570949	2.321226	1.56153	0.00908
ENSMUSG00000000290.13	Itgb2	2.2164738	20.15761	1.55998	0.00986
ENSMUSG00000041417.15	Pik3r1	3.63822	12.12098	1.55667	1.94E-07
ENSMUSG00000039824.4	Myl6b	9.8011125	32.32575	1.55406	1.32E-05
ENSMUSG00000024222.17	Fkbp5	10.537858	33.77808	1.5533	3.37E-07
ENSMUSG00000059456.13	Ptk2b	1.0142603	4.800566	1.55207	0.00636
ENSMUSG00000031827.13	Cotl1	8.49309	49.36321	1.55188	0.00819
ENSMUSG00000055612.15	Cdca7	0.7373447	4.502077	1.55025	0.00768
ENSMUSG00000115338.2	Pnp	6.46444	20.86541	1.54932	0.00057
ENSMUSG00000018008.8	Cyth4	1.3154333	5.105648	1.54853	0.0024

ENSMUSG00000040699.13	Limd2	3.399995	16.39129	1.54852	0.00194
ENSMUSG00000047945.6	Marcksl1	1.7695398	7.846978	1.54365	0.00467
ENSMUSG00000036304.14	Zdhhc23	1.5469808	5.35427	1.54295	6.93E-07
ENSMUSG00000043263.13	Ifi209	0.1618553	1.262294	1.54173	0.0091
ENSMUSG00000027078.14	Ube2l6	4.9274425	19.63027	1.54114	0.00133
ENSMUSG00000015355.13	Cd48	0.8952748	3.440689	1.53524	0.00672
ENSMUSG00000066800.11	Rnasel	0.4583185	2.234354	1.53392	0.00749
ENSMUSG00000051439.7	Cd14	0.987085	3.882865	1.53267	0.00372
ENSMUSG00000025236.11	Adpgk	4.2693625	23.95206	1.52785	0.00917
ENSMUSG00000062328.8	Rpl17	3.197825	10.25684	1.52624	0.00065
ENSMUSG00000028717.12	Tal1	0.6403828	2.977267	1.52376	0.00634
ENSMUSG00000028312.19	Smc2	0.2825295	1.549156	1.51652	0.00978
ENSMUSG00000037020.16	Wdr62	0.4369355	2.761305	1.51625	0.00059
ENSMUSG00000036246.14	Gmip	1.509397	6.702247	1.51602	0.00607
ENSMUSG00000035561.5	Aldh1b1	0.305563	1.263071	1.51591	0.00201
ENSMUSG00000074342.3	I830077J02Rik	0.3383453	2.639409	1.51509	0.00994
ENSMUSG00000049871.14	Nlrc3	0.3242995	1.215718	1.51182	0.00095
ENSMUSG00000060216.15	Arrb2	6.58948	29.41849	1.51138	0.00963
ENSMUSG00000043008.9	Klhl6	0.9968855	4.436772	1.50935	0.0067
ENSMUSG00000029467.15	Atp2a2	48.489275	154.8005	1.50585	2.79E-05
ENSMUSG00000024087.4	Cyp1b1	0.2563308	0.914737	1.50557	0.00181
ENSMUSG00000053310.11	Nrgn	1.386867	6.780115	1.49973	0.00752
ENSMUSG00000044811.13	Cd300c2	0.440032	1.751566	1.49777	0.00839
ENSMUSG00000052397.8	Ezr	1.1614535	5.131792	1.49745	0.00629
ENSMUSG00000013707.3	Tnfaip8l2	1.5465675	6.012267	1.49436	0.00755
ENSMUSG00000037411.10	Serpine1	1.4602025	4.085028	1.49227	1.38E-07
ENSMUSG00000020893.17	Per1	3.3991925	9.992948	1.48645	1.94E-10
ENSMUSG00000025747.12	Tyms	1.4753015	7.225443	1.47995	0.00904
ENSMUSG00000040009.6	Gnaz	0.1817463	0.943582	1.47812	0.00901
ENSMUSG00000068335.6	Dok1	1.2403298	3.335494	1.47357	0.0022
ENSMUSG00000002458.13	Rgs19	4.22443	15.59169	1.47264	0.00585
ENSMUSG00000001763.14	Tspan33	1.7911125	7.234348	1.4716	0.00421
ENSMUSG00000019139.10	Isyna1	2.02043	6.063588	1.46733	0.00119
ENSMUSG00000024691.13	Fam111a	1.1102025	4.692517	1.46705	0.00842
ENSMUSG00000027940.18	Tpm3	167.89153	537.3278	1.46665	7.38E-05
ENSMUSG00000003644.17	Rps6ka1	3.48861	13.10153	1.46463	0.00294
ENSMUSG00000020733.3	Slc9a3r1	1.7152525	6.841325	1.45934	0.0046
ENSMUSG00000021948.17	Prkcd	2.905865	10.60006	1.45876	0.00372
ENSMUSG00000027999.15	Pla2g12a	36.23885	105.5355	1.45821	9.96E-11

ENSMUSG00000031103.12	Elf4	0.5003488	1.452562	1.45774	0.00287
ENSMUSG00000001082.12	Mfsd10	2.3686525	8.56841	1.45304	0.00169
ENSMUSG00000040466.16	Blvrb	8.2008825	29.37375	1.45143	0.0023
ENSMUSG00000051314.11	Ffar2	0.5490788	3.134033	1.44684	0.00892
ENSMUSG00000000318.16	Clec10a	2.17972	7.357563	1.44672	3.51E-05
ENSMUSG00000042734.6	Ttc9	1.4383253	4.373035	1.44439	0.0003
ENSMUSG00000036599.10	Chst12	2.7229925	8.44546	1.44122	0.00068
ENSMUSG00000058297.16	Spock2	1.174835	3.612078	1.43396	1.24E-06
ENSMUSG00000030047.14	Arhgap25	0.681178	2.893905	1.4305	0.0074
ENSMUSG00000040283.14	Btnl9	0.452765	1.358755	1.42953	1.94E-06
ENSMUSG00000028116.13	Myoz2	95.846975	270.6105	1.4292	1.47E-06
ENSMUSG00000056201.8	Cfl1	56.821475	177.4708	1.4235	0.0011
ENSMUSG00000022489.6	Pde1b	0.3185705	1.161695	1.42118	0.00532
ENSMUSG00000022226.6	Mcpt2	1.2231155	3.704165	1.42044	0.0015
ENSMUSG00000007041.9	Clic1	10.00557	29.91048	1.42002	0.00051
ENSMUSG00000062210.13	Tnfaip8	2.9328875	8.724368	1.41772	0.00319
ENSMUSG00000034708.11	Grn	15.764725	44.88878	1.41494	5.02E-07
ENSMUSG00000026656.15	Fcgr2b	2.059905	5.921663	1.40993	0.0017
ENSMUSG00000020253.15	Ppm1m	2.7000475	9.59912	1.40845	0.0054
ENSMUSG00000022575.5	Gsdmd	2.1279675	8.093805	1.40785	0.00214
ENSMUSG00000018927.3	Ccl6	8.4731375	26.48212	1.40236	0.00168
ENSMUSG00000030878.11	Cdr2	1.6770025	5.100108	1.40157	0.00196
ENSMUSG00000034165.16	Ccnd3	15.08215	49.5097	1.39968	0.0025
ENSMUSG00000056724.15	Nbeal2	1.26859	3.752637	1.39706	0.00472
ENSMUSG00000002603.15	Tgfb1	3.94617	12.53597	1.39555	0.00336
ENSMUSG00000047181.12	Samd14	0.8712308	3.146764	1.39539	0.00665
ENSMUSG00000020303.2	Stc2	0.4888898	1.801545	1.39468	0.00046
ENSMUSG00000027077.7	Smtnl1	37.572	106.6661	1.39134	3.76E-07
ENSMUSG00000055805.15	Fmnl1	2.4357575	9.443965	1.38798	0.00706
ENSMUSG00000023067.14	Cdkn1a	7.884825	20.98648	1.38296	8.63E-08
ENSMUSG00000027947.11	Il6ra	0.6059108	1.8067	1.37828	0.00114
ENSMUSG00000038642.10	Ctss	4.4374775	15.17834	1.37805	0.0019
ENSMUSG00000034595.17	Ppp1r18	4.2895725	15.88706	1.37205	0.00488
ENSMUSG00000022346.15	Myc	1.7866475	4.944363	1.37188	0.00167
ENSMUSG00000036948.17	BC037034	2.1976525	7.727548	1.3709	0.0051
ENSMUSG00000030748.9	Il4ra	1.5804825	4.4318	1.37062	7.40E-05
ENSMUSG00000048489.12	Depp1	7.49558	24.09288	1.37015	0.00018
ENSMUSG00000001739.14	Cldn15	2.2304575	8.078693	1.36881	0.00067
ENSMUSG00000059555.6	Tor4a	0.7760585	2.222991	1.36748	0.00667

ENSMUSG00000037012.18	Hk1	2.475705	7.288835	1.35698	0.00211
ENSMUSG00000071637.5	Cebpd	4.9152875	14.01543	1.35205	3.10E-05
ENSMUSG00000022041.10	Chrna2	0.2203246	0.873588	1.351	0.00919
ENSMUSG00000029086.15	Prom1	1.6712865	5.154023	1.35036	0.00459
ENSMUSG00000007033.4	Hspa1l	4.8052875	13.5435	1.34877	1.74E-08
ENSMUSG00000029860.16	Zyx	11.9658	34.15785	1.3485	0.00127
ENSMUSG00000032060.10	Cryab	1001.7045	2827.635	1.34745	1.05E-05
ENSMUSG00000037243.17	Zfp692	2.0275225	4.35024	1.33489	0.00014
ENSMUSG00000026177.11	Slc11a1	0.700968	2.046785	1.33256	0.00722
ENSMUSG00000037902.18	Sirpa	3.2682875	10.81916	1.33207	0.00258
ENSMUSG00000046275.2	Trarg1	0.734115	2.144263	1.33164	0.00116
ENSMUSG00000007080.14	Pole	0.8395138	2.62101	1.32675	0.00256
ENSMUSG00000025422.9	Agap2	0.3777288	1.156015	1.32492	0.00161
ENSMUSG00000028656.14	Cap1	11.532868	33.05707	1.31703	0.00261
ENSMUSG00000024818.15	Slc25a45	0.923473	2.407268	1.31508	0.00798
ENSMUSG00000053166.14	Cdh22	0.550426	1.534074	1.31002	0.00189
ENSMUSG00000029683.7	Lmod2	42.388425	100.7033	1.30957	1.26E-10
ENSMUSG00000016024.9	Lbp	2.5772325	7.11745	1.3084	0.00082
ENSMUSG00000031093.14	Dock11	0.660905	2.319723	1.30294	0.00611
ENSMUSG00000004035.12	Gstm7	1.94995	4.773365	1.29802	0.00027
ENSMUSG00000043424.10	Eif3j2	2.3248435	6.767795	1.2953	0.0084
ENSMUSG00000032231.14	Anxa2	32.5321	81.77595	1.29386	8.06E-06
ENSMUSG00000033825.10	Tpsb2	1.2504473	3.84405	1.29313	0.00266
ENSMUSG00000036908.17	Unc93b1	4.9431125	14.70134	1.29169	0.00739
ENSMUSG00000025041.17	Nt5c2	1.4638755	4.14098	1.28661	1.34E-06
ENSMUSG00000021115.15	Vrk1	1.5593133	5.877588	1.28344	0.00534
ENSMUSG00000034006.17	Pqlc1	9.13469	21.77325	1.28096	4.26E-12
ENSMUSG00000004099.16	Dnmt1	1.5324225	4.766625	1.2757	0.00483
ENSMUSG00000042485.7	Mustn1	44.7191	126.729	1.27053	0.00498
ENSMUSG00000019066.13	Rab3d	1.59464	4.851203	1.26229	0.00819
ENSMUSG00000033196.17	Myh2	177.21425	433.4783	1.25999	8.49E-07
ENSMUSG00000015889.8	Lta4h	15.7582	46.36423	1.25852	0.00678
ENSMUSG00000004677.17	Myo9b	1.63915	4.148123	1.25816	0.00132
ENSMUSG00000009687.14	Fxyd5	12.740495	40.10005	1.25564	0.00517
ENSMUSG00000044456.16	Rin3	2.496325	7.27016	1.25186	0.00392
ENSMUSG00000030214.7	Plbd1	7.2171225	19.3223	1.23761	0.00283
ENSMUSG00000030747.5	Dgat2	16.646625	39.72488	1.2376	4.89E-06
ENSMUSG00000040659.3	Efhd2	5.4719625	15.11981	1.23426	0.00633
ENSMUSG00000026547.15	Tagln2	21.670175	52.635	1.22851	0.00069

ENSMUSG00000021025.8	Nfkbia	15.3434	31.0642	1.22252	1.84E-05
ENSMUSG00000007659.18	Bcl2l1	6.8750375	16.27258	1.22025	2.04E-06
ENSMUSG00000040212.12	Emp3	14.004383	36.7845	1.21516	0.00229
ENSMUSG00000005142.10	Man2b1	3.629645	8.60681	1.21258	0.00351
ENSMUSG00000029922.15	Mkrn1	9.5615925	25.6372	1.20777	0.00552
ENSMUSG00000031453.16	Rasa3	2.72727	6.564248	1.20275	0.00614
ENSMUSG00000050675.7	Gp1ba	0.9870795	2.630118	1.20219	0.00824
ENSMUSG00000074886.11	Grk6	3.9488525	10.36492	1.20198	0.00393
ENSMUSG00000040940.18	Arhgef1	11.045175	28.49678	1.1995	0.00194
ENSMUSG00000011256.16	Adam19	0.4895243	1.293219	1.19437	0.00457
ENSMUSG00000001525.10	Tubb5	19.43965	48.0414	1.19207	0.00348
ENSMUSG00000073982.11	Rhog	14.9353	39.17893	1.19196	0.00339
ENSMUSG00000002831.13	Plin4	20.460325	48.25588	1.18655	1.35E-09
ENSMUSG00000028693.15	Nasp	3.743185	9.169223	1.18577	0.00192
ENSMUSG00000053898.12	Ech1	230.028	601.8983	1.18551	1.00E-05
ENSMUSG00000015850.11	Adamtsl4	4.88631	11.30359	1.18498	1.88E-09
ENSMUSG00000027889.17	Ampd2	1.4908675	4.392958	1.17369	0.00903
ENSMUSG00000030539.13	Sema4b	0.510202	1.212321	1.17267	0.00163
ENSMUSG00000019082.18	Slc25a22	5.046645	11.60632	1.17191	2.67E-07
ENSMUSG00000060036.14	Rpl3	34.4417	85.03975	1.17118	0.00117
ENSMUSG00000033066.15	Gas7	0.9123623	2.044163	1.15649	0.00014
ENSMUSG00000030536.10	Iqgap1	2.646365	6.336293	1.15362	0.00778
ENSMUSG00000020766.4	Galk1	2.082285	5.04508	1.15229	0.00454
ENSMUSG00000048756.11	Foxo3	2.3866825	5.3032	1.15205	1.73E-06
ENSMUSG00000044167.6	Foxo1	1.397265	3.24517	1.14299	1.64E-07
ENSMUSG00000035673.10	Sbno2	1.611715	4.235073	1.14023	0.00709
ENSMUSG00000027318.17	Adam33	0.557742	1.87115	1.13617	0.00619
ENSMUSG00000026790.19	Odf2	1.865605	4.51101	1.13375	0.00253
ENSMUSG00000037447.16	Arid5a	1.54746	4.410068	1.13245	2.18E-05
ENSMUSG00000025145.13	Lrrc45	1.258308	3.14467	1.13048	0.00075
ENSMUSG00000073684.13	Faap20	3.35917	7.209843	1.1303	0.00102
ENSMUSG00000075415.13	Fnbp1	1.3706425	2.823028	1.12956	0.0021
ENSMUSG00000020057.2	Dram1	1.2766723	3.321435	1.12474	0.00708
ENSMUSG00000001025.8	S100a6	98.14445	252.0405	1.1214	0.00185
ENSMUSG00000048058.17	Ldlrad3	1.329722	3.251103	1.11906	0.00151
ENSMUSG00000019122.8	Ccl9	1.96659	4.986135	1.11901	0.00762
ENSMUSG00000020476.14	Dbnl	6.28548	15.50051	1.11648	0.00283
ENSMUSG00000056737.14	Capg	10.280408	24.85683	1.11435	0.00026
ENSMUSG00000022540.16	Rogdi	5.4068625	11.78781	1.11384	0.00172

ENSMUSG00000025283.15	Sat1	15.001425	33.68593	1.11288	0.00156
ENSMUSG00000040618.7	Pck2	1.9995	5.304185	1.11139	0.0062
ENSMUSG00000032624.16	Eml4	0.470543	1.152427	1.111	0.00765
ENSMUSG00000057110.15	Cntrl	1.456665	4.510973	1.10887	0.00555
ENSMUSG00000022206.7	Npr3	1.127469	3.085728	1.10476	0.00297
ENSMUSG00000021750.15	Fam107a	1.1345688	2.416143	1.10207	5.95E-05
ENSMUSG00000021255.17	Esrrb	1.4256625	2.919375	1.10062	0.00021
ENSMUSG00000026849.18	Tor1a	3.175145	7.175743	1.09976	0.00796
ENSMUSG00000063511.11	Snrnp70	21.1454	48.72068	1.09959	5.06E-08
ENSMUSG00000049907.8	Rasl11b	1.5550475	3.817375	1.09832	0.0014
ENSMUSG00000039616.10	Mocos	0.7785833	1.962238	1.08703	0.00224
ENSMUSG00000025375.15	Aatk	1.1980243	2.471128	1.08391	0.00126
ENSMUSG00000041736.7	Tspo	42.808825	100.557	1.0796	0.00887
ENSMUSG00000024164.15	C3	10.817458	26.37398	1.0782	0.00618
ENSMUSG00000031167.16	Rbm3	22.8698	50.0727	1.07418	0.00127
ENSMUSG00000017631.18	Abr	2.3940925	5.04936	1.07317	0.00011
ENSMUSG00000056515.9	Rab31	2.1054225	4.579913	1.07297	0.00869
ENSMUSG00000085795.8	Zfp703	8.725685	19.53215	1.07129	1.65E-06
ENSMUSG00000003123.15	Lipe	9.28977	19.84895	1.07121	4.92E-05
ENSMUSG00000022555.12	Dgat1	12.30265	26.22421	1.06633	0.00242
ENSMUSG00000059741.13	Myl3	414.17175	957.1393	1.05663	0.00741
ENSMUSG00000001918.17	Slc1a5	6.819635	13.86394	1.05504	0.0013
ENSMUSG00000036585.16	Fgf1	5.9434175	12.25721	1.05151	3.88E-05
ENSMUSG00000024063.13	Lbh	3.03676	6.239448	1.04575	0.00023
ENSMUSG00000018822.7	Sfrp5	3.4388125	8.08346	1.04525	0.00886
ENSMUSG00000022018.7	Rgcc	44.319675	93.6578	1.03991	2.69E-05
ENSMUSG00000056629.16	Fkbp2	11.526443	24.84215	1.0394	0.00043
ENSMUSG00000071379.2	Hpcal1	4.5653425	9.452443	1.03911	0.00845
ENSMUSG00000074918.4	Inafm2	1.636585	3.445238	1.03289	0.00543
ENSMUSG00000030541.16	Idh2	163.831	330.9155	1.02547	1.31E-05
ENSMUSG00000035493.10	Tgfbi	7.448065	16.47529	1.02438	0.00595
ENSMUSG00000096472.2	Cdkn2d	10.765265	24.8794	1.02359	0.00866
ENSMUSG00000042770.8	Hebp1	8.12911	17.24461	1.01944	0.00834
ENSMUSG00000084349.3	Rpl3-ps1	8.482915	18.06358	1.01743	0.00463
ENSMUSG00000039168.15	Dap	6.6178575	13.86723	1.01632	0.00223
ENSMUSG00000025085.16	Ablim1	5.09845	11.05544	1.01265	0.00041
ENSMUSG00000010461.15	Eya4	4.9142575	2.27685	-1.0029	9.94E-05
ENSMUSG00000038418.7	Egr1	11.254578	4.817758	-1.0154	0.00947
ENSMUSG00000073139.9	Tmem185a	17.139175	7.62312	-1.0234	4.28E-09

ENSMUSG00000028270.12	Gbp2	3.94217	1.827743	-1.0259	0.00122
ENSMUSG00000028836.14	Slc30a2	5.4935775	2.417818	-1.0272	0.00934
ENSMUSG00000039405.7	Prss23	41.671675	19.04868	-1.0282	4.91E-05
ENSMUSG00000020546.14	Stxbp4	3.1237225	1.5341	-1.0331	7.65E-06
ENSMUSG00000027559.5	Car3	1684.9675	744.6558	-1.0417	0.00586
ENSMUSG00000070803.6	Cited4	18.123625	8.509118	-1.0491	0.00419
ENSMUSG00000002012.13	Pnck	10.206173	4.405593	-1.0537	0.00042
ENSMUSG00000056973.6	Ces1d	29.79715	14.05335	-1.0571	0.00368
ENSMUSG00000078202.3	Nrarp	4.32106	1.76518	-1.0618	0.00226
ENSMUSG00000035606.8	Ky	33.958225	15.59204	-1.0696	1.03E-05
ENSMUSG00000038248.8	Sobp	6.793425	3.17717	-1.0744	6.73E-06
ENSMUSG00000042717.5	Ppp1r3a	30.4838	13.9709	-1.0785	1.12E-05
ENSMUSG00000062937.7	Mtap	7.6627425	3.800188	-1.0798	6.30E-08
ENSMUSG00000027776.12	Il12a	9.7751775	3.037555	-1.0951	0.00088
ENSMUSG00000062785.14	Kcnc3	0.9684825	0.308947	-1.0971	0.00508
ENSMUSG00000053205.9	Styx	7.255905	1.93597	-1.1037	2.57E-09
ENSMUSG00000060639.5	Hist1h4i	35.030575	15.59833	-1.1084	0.00194
ENSMUSG00000021930.14	Spryd7	16.70665	6.861475	-1.1104	3.53E-05
ENSMUSG00000026827.12	Gpd2	33.85795	15.28325	-1.1167	1.63E-08
ENSMUSG00000028630.9	Dyrk2	9.0820675	4.301088	-1.1215	5.68E-06
ENSMUSG00000055435.6	Maf	29.87895	13.80188	-1.1277	4.30E-08
ENSMUSG00000045246.11	Kcng4	21.693125	10.15557	-1.1307	9.42E-08
ENSMUSG00000025586.17	Cpeb1	8.0953975	3.30361	-1.1338	1.66E-05
ENSMUSG00000035133.9	Arhgap5	6.3765625	2.853175	-1.1445	0.00033
ENSMUSG00000031885.14	Cbfb	36.969325	16.96058	-1.1501	1.36E-08
ENSMUSG00000019768.16	Esr1	4.997905	2.10608	-1.1552	1.21E-07
ENSMUSG00000064339.1	mt-Rnr2	1274.3725	551.5143	-1.1607	8.05E-09
ENSMUSG00000004360.9	9330159F19Rik	4.1735575	1.652675	-1.1622	5.28E-05
ENSMUSG00000027895.9	Kcnc4	40.417025	16.9943	-1.1679	3.68E-05
ENSMUSG00000044951.15	Mylk4	221.2625	88.06613	-1.1718	0.00011
ENSMUSG00000022003.7	Slc25a30	2.9935675	1.232198	-1.1728	0.00017
ENSMUSG00000032883.15	Acsl3	5.6089825	2.55174	-1.1764	1.66E-05
ENSMUSG00000030306.14	Tmtc1	12.0583	5.18675	-1.1875	1.73E-09
ENSMUSG00000020538.15	Srebf1	16.87685	7.42052	-1.199	1.70E-07
ENSMUSG00000081194.1	Gm8424	4.301965	1.372147	-1.2072	0.00816
ENSMUSG00000026107.11	Nabp1	4.924075	2.014858	-1.21	0.00013
ENSMUSG00000035735.10	Dagla	2.287855	0.950568	-1.2296	2.24E-05
ENSMUSG00000026399.12	Cd55	26.732375	11.08501	-1.2334	6.06E-09
ENSMUSG00000047963.7	Stbd1	46.127	18.32325	-1.2486	1.94E-08

ENSMUSG00000054072.12	Iigp1	6.2572325	2.13398	-1.2516	0.00079
ENSMUSG00000030102.11	Itpr1	2.7251875	1.147551	-1.2623	3.82E-08
ENSMUSG00000064337.1	mt-Rnr1	1634.345	652.5235	-1.271	1.63E-09
ENSMUSG00000038205.12	Prkab2	81.603125	31.92645	-1.2892	6.00E-08
ENSMUSG00000094786.1	Gm14403	4.12255	1.192494	-1.2908	0.00901
ENSMUSG00000063821.6	Dupd1	40.919875	13.39386	-1.3275	0.00432
ENSMUSG00000039304.11	Tnfsf10	1.48266	0.574304	-1.3288	3.71E-05
ENSMUSG00000051043.16	Gprc5c	15.0339	5.234053	-1.3509	7.78E-07
ENSMUSG00000019932.8	Kera	3.792175	1.124656	-1.3565	0.00125
ENSMUSG00000050014.8	Apol10b	2.211285	0.80514	-1.3602	9.37E-05
ENSMUSG00000026100.6	Mstn	17.099975	5.91325	-1.361	1.78E-05
ENSMUSG00000046828.3	Mettl21e	20.7135	7.50046	-1.373	2.31E-06
ENSMUSG00000031099.16	Smarca1	2.38451	0.711669	-1.3857	1.49E-05
ENSMUSG00000107330.2	Ppp2r5b	0.8297583	0.250241	-1.3978	0.00311
ENSMUSG00000036526.8	Card11	3.2532075	0.965872	-1.4009	0.00071
ENSMUSG00000026773.19	Pfkfb3	20.24	7.729575	-1.4142	9.85E-10
ENSMUSG00000032076.19	Cadm1	1.8276025	0.556743	-1.4302	3.41E-05
ENSMUSG00000036902.11	Neto2	0.713736	0.208338	-1.4386	0.00367
ENSMUSG00000000317.11	Bcl6b	2.2814725	0.736454	-1.4598	0.00029
ENSMUSG00000062488.9	Ifit3b	2.79098	0.851759	-1.4722	0.00023
ENSMUSG00000055214.15	Pld5	0.7355398	0.22806	-1.4802	0.00277
ENSMUSG00000068614.7	Actc1	1200.797	240.6641	-1.4891	0.00943
ENSMUSG00000051980.13	Casr	1.483206	0.411615	-1.4917	0.00092
ENSMUSG00000027313.3	Chac1	30.43905	9.431758	-1.4955	1.04E-06
ENSMUSG00000026185.8	Igfbp5	115.08285	38.3495	-1.5228	2.96E-10
ENSMUSG00000028444.17	Cntfr	16.7674	5.587793	-1.5321	3.86E-09
ENSMUSG00000020169.4	Best3	1.2955605	0.447377	-1.534	6.01E-05
ENSMUSG00000055980.2	Irs1	2.684235	0.538189	-1.5585	2.99E-09
ENSMUSG00000022591.5	Gm9747	20.070725	7.247525	-1.5659	9.90E-15
ENSMUSG00000037010.7	Apln	0.841618	0.24328	-1.5793	0.00017
ENSMUSG00000050069.3	Grem2	4.04355	1.127732	-1.5987	0.00023
ENSMUSG00000032313.11	Tmem266	1.6930675	0.500716	-1.611	1.03E-05
ENSMUSG00000020027.18	Socs2	4.201595	1.402173	-1.6394	1.57E-07
ENSMUSG00000057666.18	Gapdh	170.30023	20.9276	-1.6404	0.00052
ENSMUSG00000027333.18	Smox	243.2353	65.99773	-1.6471	0.00169
ENSMUSG00000025777.8	Gdap1	2.9192425	0.87252	-1.6904	4.47E-09
ENSMUSG00000068606.6	Gm4841	4.98802	1.424282	-1.7218	4.31E-05
ENSMUSG00000048939.13	Atp13a5	0.7038693	0.2249	-1.7287	2.14E-05
ENSMUSG00000076617.9	Ighm	160.58608	39.56328	-1.7325	7.42E-05

ENSMUSG00000044338.9	Aplnr	1.6189	0.404021	-1.7549	1.62E-06
ENSMUSG00000040253.15	Gbp7	1.3958758	0.280103	-1.7663	1.54E-05
ENSMUSG00000063388.5	BC023105	16.2233	4.085918	-1.8314	2.00E-06
ENSMUSG00000074634.12	Tmem267	3.1493895	0.550754	-1.834	0.00072
ENSMUSG00000021668.15	Polk	2.4903075	0.563002	-1.8381	7.80E-11
ENSMUSG00000079491.9	H2-T10	8.9840725	2.479418	-1.8471	1.32E-05
ENSMUSG00000070498.3	Tmem132b	0.698008	0.111939	-1.8722	0.00023
ENSMUSG00000042834.15	Nrep	84.456625	18.22485	-1.9444	4.63E-08
ENSMUSG00000025887.10	Casp12	4.720765	1.228905	-2.0491	2.13E-09
ENSMUSG00000055003.14	Lrtm2	0.8546326	0.103141	-2.1512	0.00014
ENSMUSG00000090942.1	F830016B08Rik	1.3831578	0.201865	-2.1989	1.06E-05
ENSMUSG00000051985.12	Igfn1	10.781473	1.96069	-2.2524	4.63E-07
ENSMUSG00000075232.5	Amd1	143.9873	23.3427	-2.4044	2.01E-12
ENSMUSG00000060096.5	Amd-ps3	9.536525	1.485423	-2.4177	2.03E-09
ENSMUSG00000025479.9	Cyp2e1	4.37487	0.614454	-2.4241	6.50E-09
ENSMUSG00000041460.14	Cacna2d4	0.7438723	0.095702	-2.5325	4.64E-07
ENSMUSG00000066407.3	Gm10263	44.47735	1.782748	-2.5495	1.47E-06
ENSMUSG00000063953.3	Amd2	11.639383	1.330757	-2.7832	3.47E-11
ENSMUSG00000062611.4	Rps3a2	43.880763	1.65717	-2.9495	2.83E-08
ENSMUSG00000027827.16	Kcnab1	2.0560903	0.222749	-3.0318	8.92E-10
ENSMUSG00000044320.14	1700001O22Rik	7.0324175	0.102542	-3.3489	5.73E-09

Supplementary Table 4: Differentially regulated genes belonging to pathways of insulin resistance and insulin, FOXO and AMPK signaling and identified as potential targets to altered miRNAs in diabetic (db/db) mice

Gene name	GeneExpression	Insulin Signaling	Insulin Resistance	AMPK Signaling	FOXO Signaling	Targeting miRNAs
Prkar2b	**Up**	Y	N	N	N	miR-872-3p, miR-509-3p
Foxo1	**Up**	Y	Y	Y	Y	miR-145a-3p
Hk1	**Up**	Y	N	N	N	miR-34c-5p
Pik3r5	**Up**	Y	Y	Y	Y	miR-764-5p
Pik3r1	**Up**	Y	Y	Y	N	miR-539-5p, miR-320-3p, miR-381-3p, miR-503-5p, miR-540-5p
Nfkbia	**Up**	N	Y	N	N	miR-381-3p
Prkcd	**Up**	N	Y	N	N	miR-374b-5p
Rps6ka1	**Up**	N	Y	N	N	miR-302b-3p
Pfkfb3	**Down**	N	N	Y	N	miR-291b-3p, miR-let7c-2-3p
Cdkn2d	**Up**	N	N	N	Y	miR-539-5p
Foxo3	**Up**	N	N	Y	Y	miR-764-5p
Agap2	**Up**	N	N	N	Y	miR-34c-5p, miR-499a-5p
Tgfb1	**Up**	N	N	N	Y	miR-425-3p

N: Does not belong to the pathway indicated: Y: Belongs to the pathway indicated
